# Karyotype and LTR-RTs analysis provide insights into oak genomic evolution

**DOI:** 10.1186/s12864-024-10177-6

**Published:** 2024-04-03

**Authors:** Rui-Bin Cao, Ran Chen, Ke-Xin Liao, He Li, Gang-Biao Xu, Xiao-Long Jiang

**Affiliations:** The Laboratory of Forestry Genetics, Central South University of Forestry and Technology, 410004 Changsha, Hunan China

**Keywords:** Whole-genome duplication, Long terminal repeat retrotransposons, Karyotype, Oak, Chromosomal structure, Genomic evolution

## Abstract

**Background:**

Whole-genome duplication and long terminal repeat retrotransposons (LTR-RTs) amplification in organisms are essential factors that affect speciation, local adaptation, and diversification of organisms. Understanding the karyotype projection and LTR-RTs amplification could contribute to untangling evolutionary history. This study compared the karyotype and LTR-RTs evolution in the genomes of eight oaks, a dominant lineage in Northern Hemisphere forests.

**Results:**

Karyotype projections showed that chromosomal evolution was relatively conservative in oaks, especially on chromosomes 1 and 7. Modern oak chromosomes formed through multiple fusions, fissions, and rearrangements after an ancestral triplication event. Species-specific chromosomal rearrangements revealed fragments preserved through natural selection and adaptive evolution. A total of 441,449 full-length LTR-RTs were identified from eight oak genomes, and the number of LTR-RTs for oaks from section *Cyclobalanopsis* was larger than in other sections. Recent amplification of the species-specific LTR-RTs lineages resulted in significant variation in the abundance and composition of LTR-RTs among oaks. The LTR-RTs insertion suppresses gene expression, and the suppressed intensity in gene regions was larger than in promoter regions. Some centromere and rearrangement regions indicated high-density peaks of LTR/*Copia* and LTR/*Gypsy*. Different centromeric regional repeat units (32, 78, 79 bp) were detected on different *Q. glauca* chromosomes.

**Conclusion:**

Chromosome fusions and arm exchanges contribute to the formation of oak karyotypes. The composition and abundance of LTR-RTs are affected by its recent amplification. LTR-RTs random retrotransposition suppresses gene expression and is enriched in centromere and chromosomal rearrangement regions. This study provides novel insights into the evolutionary history of oak karyotypes and the organization, amplification, and function of LTR-RTs.

**Supplementary Information:**

The online version contains supplementary material available at 10.1186/s12864-024-10177-6.

## Background

Chromosomal mutations, such as polyploidization and chromosomal rearrangement, can lead to speciation, adaptation, and diversification [1–5]. Extant species are ancient polyploids from a common ancestor that experienced at least one whole-genome duplication (WGD) [6]. Eudicots core to their clade descended from an ancient whole-genome triplication event (*γ*) [7]. Chromosomal evolution influences the development of chromosomal size, structure, composition, and number of chromosomes [8]. Karyotype evolution will cause the chromosomal structure to be unstable, such as fusion and fission regions caused by rearrangement, as well as centromere regions that increase or disappear due to WGD or chromosome fusion [9]. Transposable elements may fill and stabilize these unstable regions in the chromosomes [10]. Therefore, reconstructing the ancestor karyotype and analysing the distribution of transposable elements are crucial for untangling the species local adaptation and speciation.

Previous approaches for ancestral karyotype reconstruction and projection defined contiguous ancestral regions based on collinearity among genomes. This method results in gaps in the projections and reveals unrefined karyotype details [11–13]. Based upon the assumption that ancestral chromosomes remain in contemporary genomes, a new method has been proposed to search shared intact chromosomes or chromosome-like syntenic blocks to construct a gap-less ancestor karyotype projection [14]. The newly constructed ancestral eudicot karyotype (AEK) and ancestral core eudicot karyotype (ACEK) would provide a better model for karyotype projections of modern species, and inform further research into the evolutionary history of Kingdom Plantae [15].

Along with polyploidization, amplification of transposable elements (TEs) is a primary form of mutation affecting the structure, function, and evolution of chromosomes [16–20]. Long terminal repeat retrotransposons (LTR-RTs) are major components of TEs in plant genomes, accounting for > 70% of the nuclear genomes of maize [21], tea [22], and rye [23]. However, their abundance and composition vary across species due to genome size and LTR-RT amplification [24, 25]. According to the positions of integrase (INT), LTR-RTs can be divided into Ty1/*Copia* and Ty3/*Gypsy* superfamilies and different lineages [26, 27]. The abundance of LTR-RTs specific-lineages is considered one of the important factors affecting species adaptation [28]. LTR-RTs spread throughout the genomes by retrotransposition (a copy/paste mechanism) during species evolution, which causes LTR-RTs amplification, genome expansion, and chromosome rearrangement [29, 30]. The LTR-RTs amplification contributes to chromosomal structure, centromere function, and regulation of gene expression [31–33]. Therefore, exploring the abundance, distribution, and evolutionary dynamics of LTR-RTs helps explain the molecular mechanism of chromosomal structural variation and evolutionary processes in genomes.

*Quercus* (oak), the largest genus in the family Fagaceae, is widely distributed in the Northern Hemisphere, including Asia, Europe, Africa, and the Americas [34]. As an important ecological and economic tree in East Asia, oaks are famous for their environmental adaptability, resistance to biotic and abiotic stresses, and providing many biological materials [35, 36]. Currently, eight chromosome-level oak genomes have been sequenced and annotated, including *Q. acutissima* [37], *Q. dentata* [38], *Q. gilva* [39], *Q. glauca* [40], *Q. lobata* [41], *Q. mongolica* [42], *Q. robur* [11], and *Q. variabilis* [43]. These provide a comprehensive database for analysing the genomic and chromosomal evolution of the genus. The evolutionary history and phylogenetic relationships of *Quercu*s are well-established using high-quality nuclear and chloroplast genomes [44, 45]. The genus dates back to approximately 55 Ma (millions of years ago), and there have been no significant levels of chromosome fusion or species-specific WGD events [46–49].

Previous comparative genomics research on *Quercus* mainly concentrated on analysing interspecies genomic collinearity, phylogenetic relationships, and demographic dynamics. The karyotype evolution and LTR-RT diversity of oak species remain unknown so far. Understanding the karyotype evolution and LTR-RT distribution is important for a comprehensive and objective view of the oak evolution. Here, based on the high-quality oak genome sequencing, we aim to (I) reveal the chromosomal evolutionary history, (II) investigate intergeneric variation and evolutionary dynamics of LTR-RTs, (III) explore the influence of LTR-RTs insertion on gene regulation, chromosomal structure, and centromere functional. This study also provides a case for exploring species adaptation evolution and speciation from the perspective of karyotype and LTR-RT evolution.

## Results

### Chromosomal evolution of oaks

**Fig. 1:**
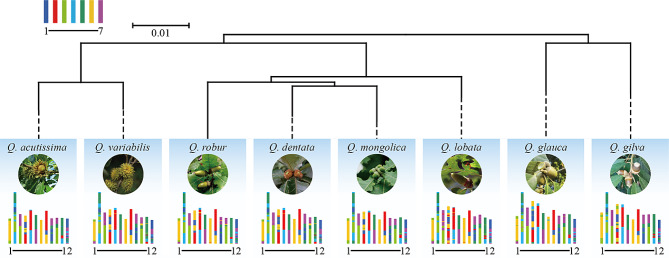
Karyotype (AEK) projections for eight oak species

To infer the chromosomal evolution of *Quercus* (2n = 24), ancestral karyotype projections were reconstructed using the AEK as a reference. Synteny blocks and gene pairs between contemporary oak genomes and the AEK were, respectively, 306–505 and 5,929 − 15,865 (Table S2). *Quercus* chromosomes 1 and 7 have a conserved synteny relationship with AEK 6, and the other ten chromosomes exhibited a fusion of synteny blocks with fragmented ancestral chromosomes (Fig. 1 and S1). For example, *Quercus* chromosome 2 showed the synteny relationship with at least 4 AEK chromosomes and 11 fragments. Homologous gene dot-plots and the karyotype projections between *Quercus* and the ACEK were completed to explore the impact of ancient triplication events on the karyotype evolution (Figs. S2 and S3). Synteny blocks and gene pairs between oak species and the ACEK were 782-1,107 and 15,865 − 20,446, respectively (Table S2). ACEK chromosomes 3, 4, 6, 10, 13, and 15 were intact and preserved in the *Quercus* genomes. Other ACEK chromosomes were preserved as fragments in different *Quercus* chromosomes. Through chromosome arm exchange, for example, the ACEK 7 is preserved in the *Quercus* chromosomes 6 and 10. Intra-chromosomal rearrangements, such as the inversion of ACEK 5 on *Quercus* chromosome 11 and ACEK 7 on *Quercus* chromosome 6, indicated complex chromosome variation during oak evolution.

**Fig. 2:**
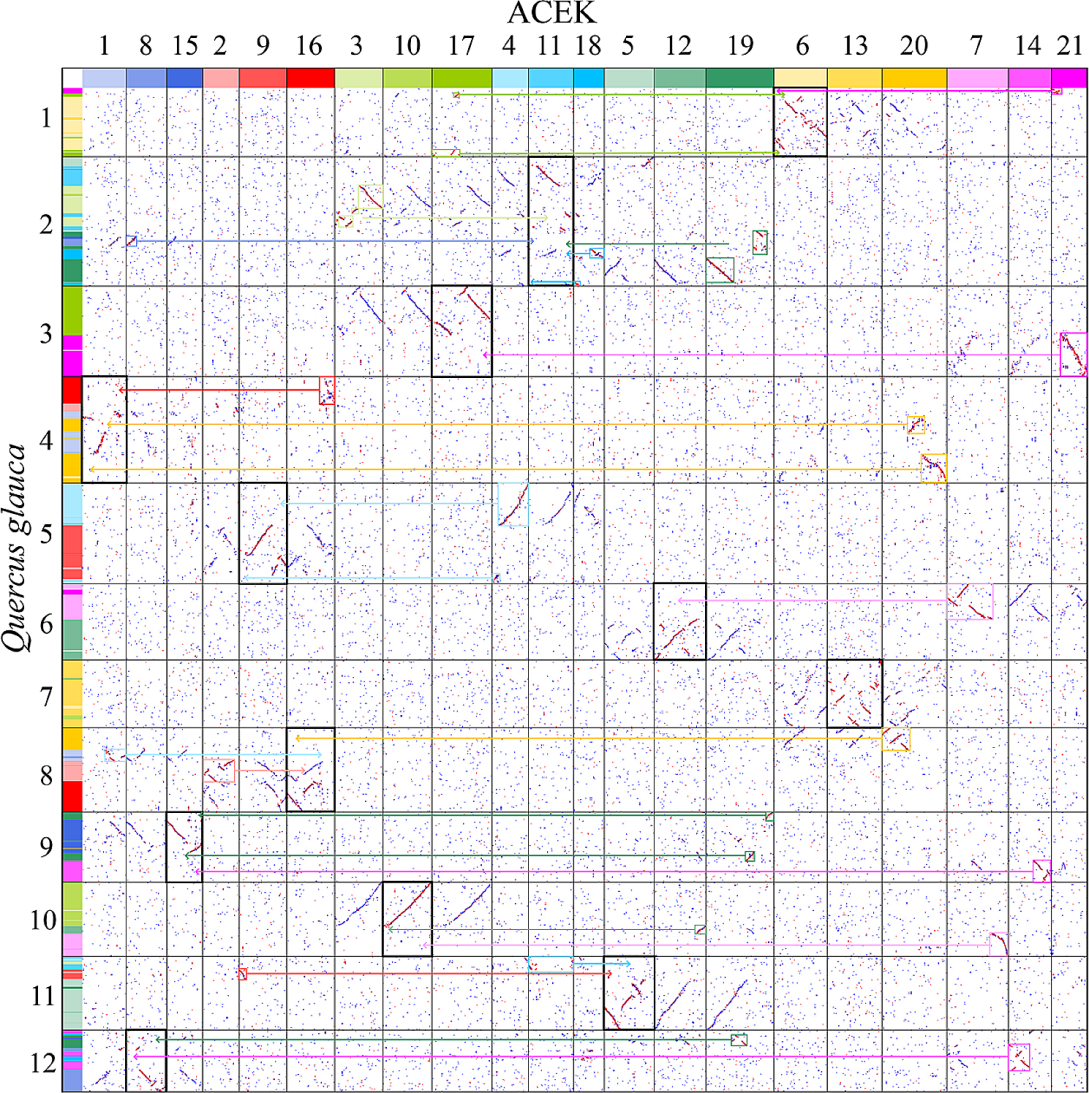
Evolution of modern chromosomes in *Q. glauca.* Arrows indicate ancestral pieces fused into one chromosome. Black boxes refer to the reference chromosomes to construct the modern karyotype of *Q. glauca*

To examine the ancestral chromosomal changes in oak species, we clarified the evolution of *Q. glauca* chromosomes. A total of six chromosomes were fused by two ACEK chromosomes. *Quercus glauca* chromosome 2 was fused by four ACEK chromosomes (ACEK 3, ACEK 11, ACEK 18, and ACEK 19; Fig. 2). Chromosome arm exchanges were observed in several chromosome pairs, such as 4 and 8, 6 and 10, and 9 and 12. After multiple chromosome fusions and arm exchanges, the chromosome number of *Q. glauca* remained stable. A total of 907-1,427 synteny blocks and 19,027 − 23,531 gene pairs were identified between *Q. glauca* and the other seven oak genomes (Table S2). Homologous gene dot-plots detected species-specific chromosomal rearrangements, such as chromosomes 1 and 7 of *Q. gilva* and chromosomes 4 and 11 of *Q. dentata* (Fig. S5). An inversion of approximately 5.6 Mb in chromosome 3 was unique to *Q. glauca*. Another inversion of chromosome 3 at ca. 7.6 Mb to 10.7 Mb and ca. 56.7 Mb to 82.4 Mb were unique in *Q. lobata*.

### Evolution of full-length LTR-RTs in Oaks

**Fig. 3:**
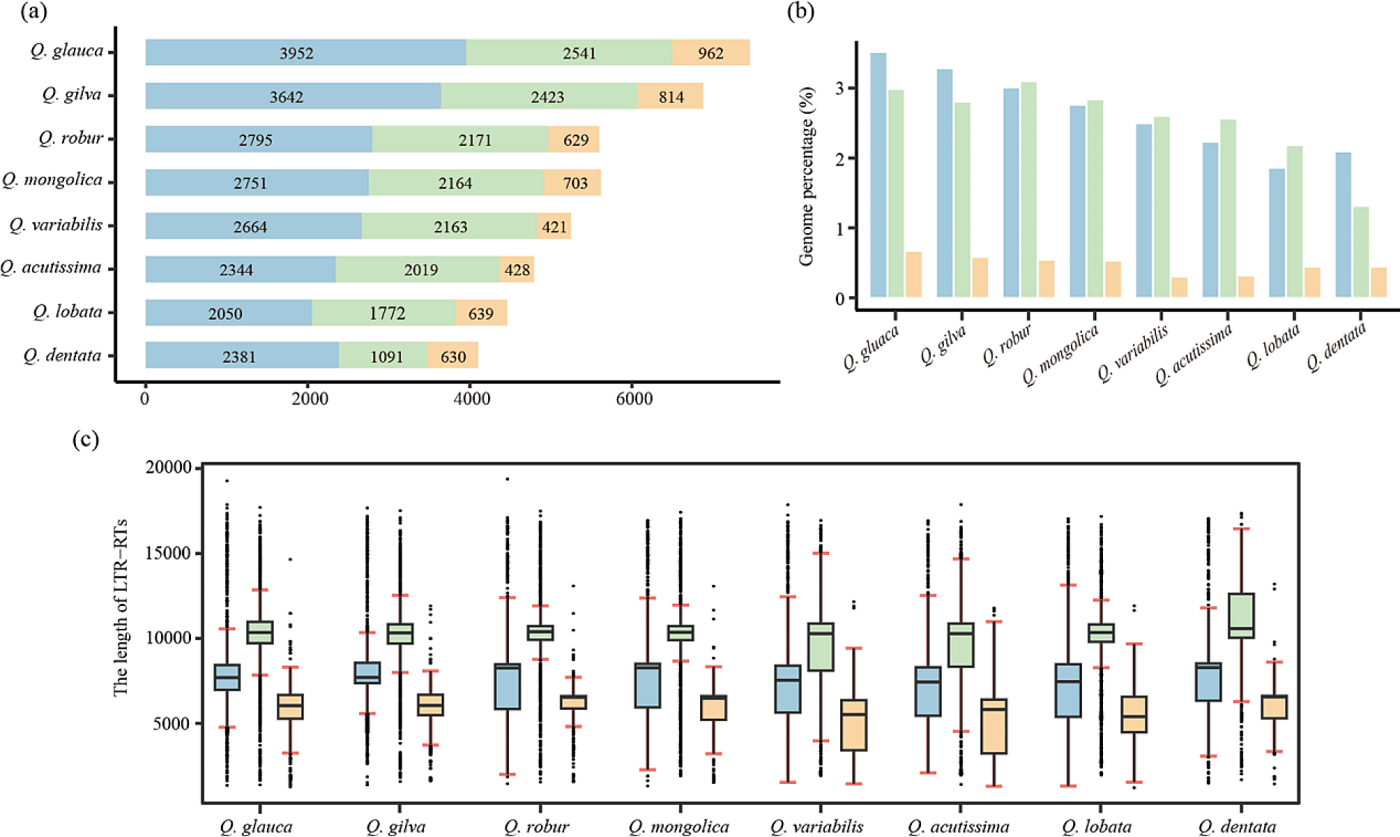
Full-length LTR-RTs number, average length, and proportions across eight oak species. Blue represents the *Copia*; Green represents the *Gypsy*; Orange represents the Unknown. **a** Number of *Copia*, *Gypsy*, and Unknown were detected in eight oak species. **b** Genome proportion of *Copia*, *Gypsy*, and Unknown of each species. **c** The average length of the full-length LTR-RTs in eight oak species

To explore the evolution of oak chromosomal structure, a total of 441,449 full-length LTR-RTs were identified in the eight genomes, including 22,579 Ty1/*Copia* (51.1%), 16,344 Ty3/*Gypsy* (37.0%), and 5,226 designated as Unknown (11.9%; Table S3). The densities (average number per Mb genome) of LTR-RTs in oak species varied from 4.6 (*Q. dentata*) to 8.6 (*Q. glauca*), and the cumulative length from 33.8 Mb (*Q. dentata*) to 56.8 Mb (*Q. gilva*; Table S3). The number of solo LTRs in the oak species varied from 83,118 (*Q. robur*) to 152,408 (*Q. dentata*), and the cumulative length from 93.2 Mb (*Q. mongolica*) to 136.7 Mb (*Q*. *dentata*; Table S3). The number of full-length LTR-RTs was variable between oak species, ranging from 4,102 (*Q. dentata*) to 7,455 (*Q. glauca*; Fig. 3a). The genomic content masked by LTR-RTs ranged from 3.8% (*Q. dentata*) to 7.1% (*Q. glauca*; Fig. 3b). In all oak species, *Copia* types were more abundant than *Gypsy*, and the average length of *Gypsy* types was larger than that of the *Copia* and Unknown (Fig. 3c).

**Fig. 4:**
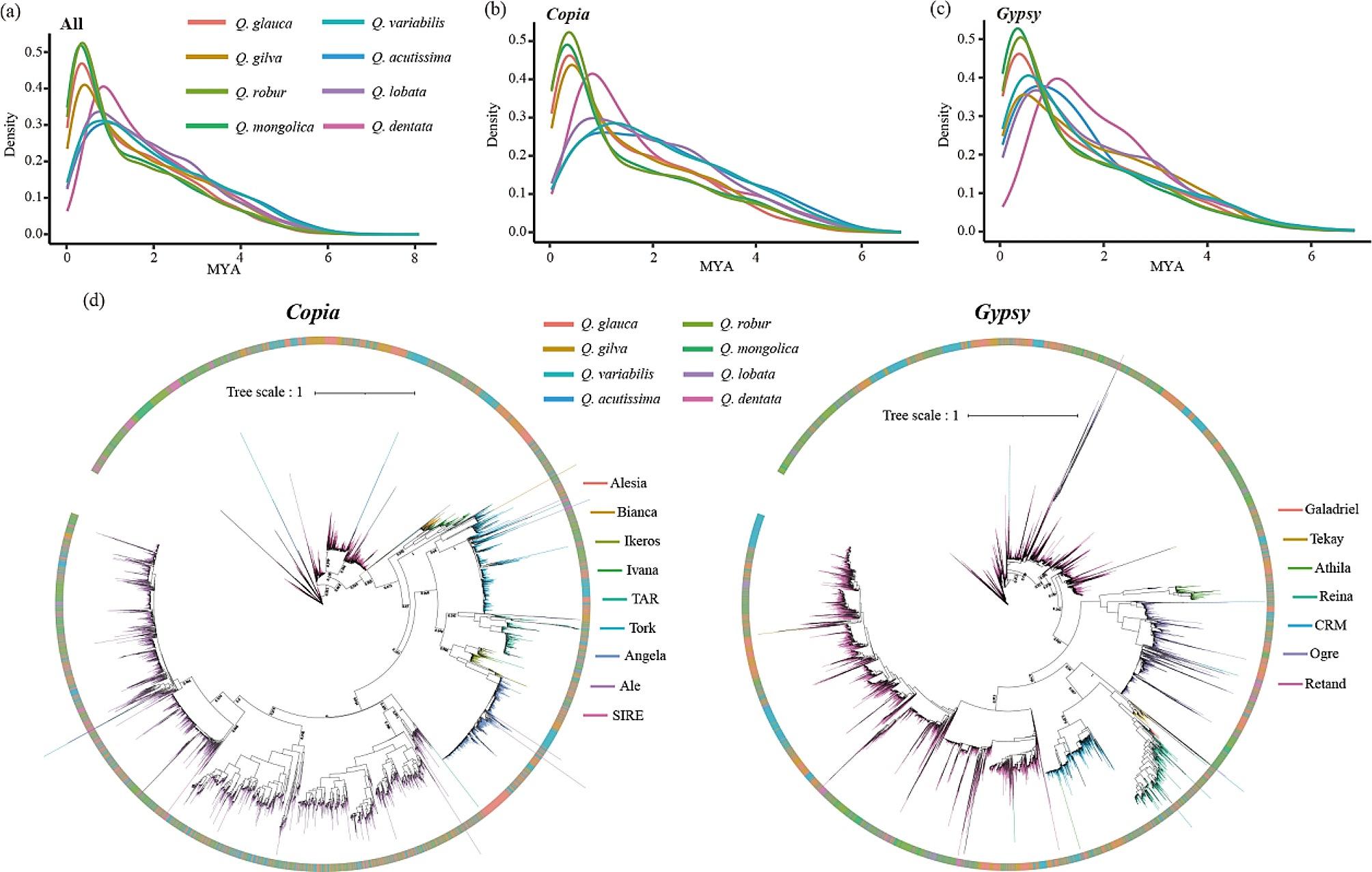
Evolution and diversity of LTR-RTs lineages in each oak species. **a** The estimated insertion time of all full-length LTR-RTs (MYA, millions of years ago); **b** The estimated insertion time of *Copia*; **c** The estimated insertion time of *Gypsy*; **d** Phylogenetic trees constructed based on reverse transcriptase domain sequences. The different colors on the outer circle represent different species, and the different colors on the Branch represent different lineages

The transposition time of LTR-RTs was estimated to be within the last 8 Ma (Fig. 4a). Four oak species (*Q. gilva*, *Q. glauca*, *Q. mongolica*, and *Q. robur*) showed more recent amplification within the last 0.2 Ma. Recent amplification of LTR-RTs in *Q. dentata* (about 0.8 Ma) was more ancient than the above four oak species. Differences in LTR-RTs amplification in oak species were mainly due to the difference in the insertion time of *Copia* (Fig. 4b, c and S6). Five species (*Q. dentata*, *Q. gilva*, *Q. glauca*, *Q. mongolica*, and *Q. robur*) showed dramatic *Copia* amplification. Two species (*Q. acutissima* and *Q. variabilis*) from section *Cerris* showed that the insertion time of *Gypsy* was more recent than that of *Copia*.

According to their RT protein domains, the *Copia* and *Gypsy* types were subclassified into nine and seven lineages, respectively (Fig. S7). In *Copia*, SIRE, Ale, and Tork lineages were most common, and Retand and Ogre lineages were most common in *Gypsy*. The maximum likelihood (ML) tree indicated that much species-specific amplification occurred for several lineages in different species (Fig. 4d). The *Copia*/Ale lineages were amplified relatively ancient in *Q. dentata* (Fig. S8a). The *Copia*/SIRE lineages showed an activity burst in five oak species (*Q. dentata*, *Q. gilva*, *Q. glauca*, *Q. mongolica*, and *Q. robur*), and the burst of *Q. dentata* was more ancient than other species (Fig. S8b). The *Copia*/Angela lineages were only abundant in four oak species (*Q. acutissima*, *Q. gilva*, *Q. glauca*, and *Q. variabilis*), and a recent more active burst in *Q. glauca* was due to the amplification of *Copia*/Angela lineages (Fig. S8c). The *Gypsy*/Retand lineages showed variation in insertion time within all eight oak species (Fig. S8d).

### Distribution of LTR-RTs in oaks

**Fig. 5:**
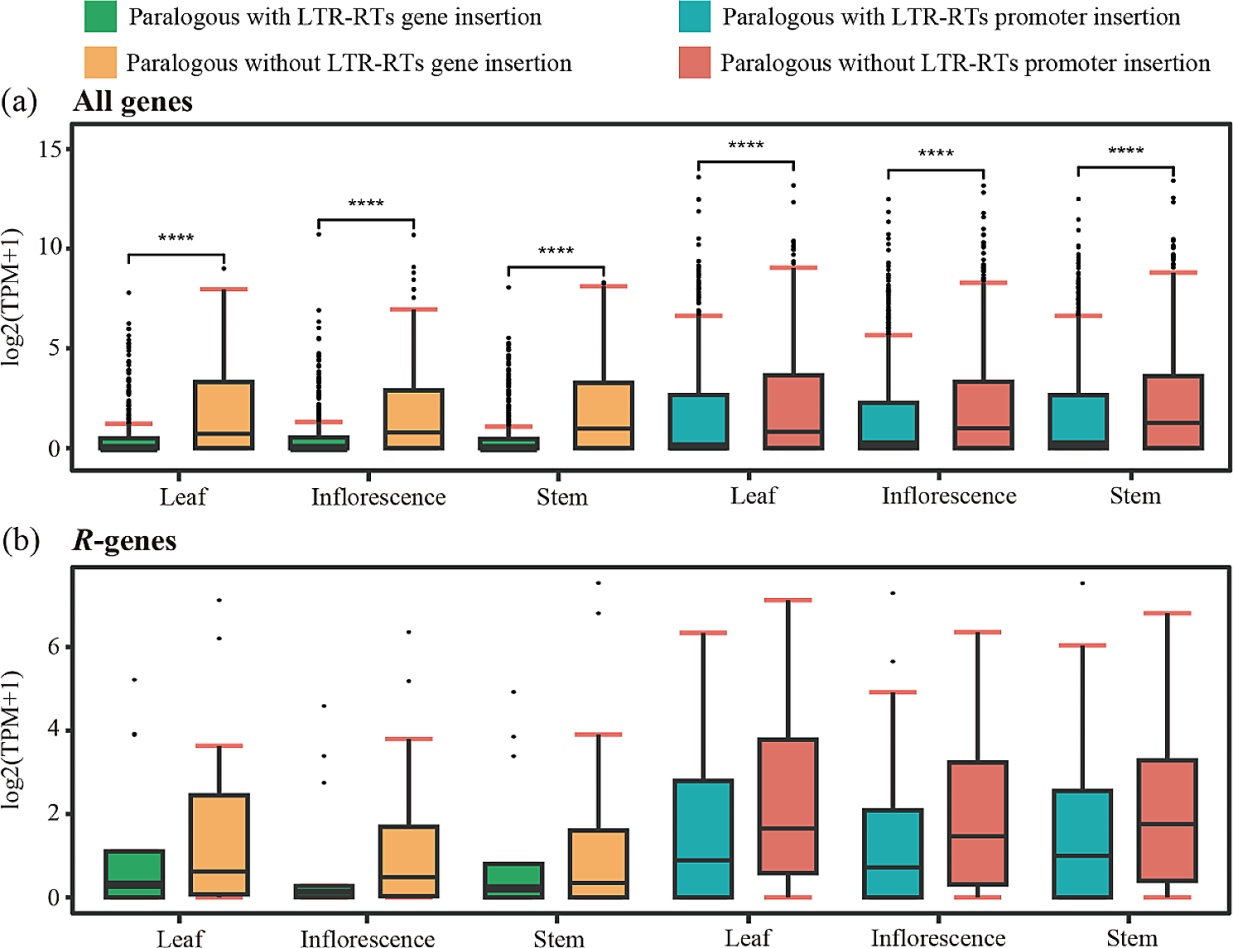
Impact of LTR-RTs on gene expression. **a** Gene expression levels of paralogous gene pairs with or without LTR-RTs insertion in three tissues of *Q. glauca*. p****<0.001. **b***R*-gene expression levels of paralogous gene pairs with or without LTR-RTs insertion

LTR-RTs are widely distributed in plant genomes through retrotransposition and may be inserted into the promoter or coding regions of genes. In oak species, we found more LTR-RTs inserted in promoter regions (293-1,772) than gene regions (302-1,495), except for in *Q. lobata* (Fig. S9 and Table S4). Inserted LTR-RTs suppressed gene expression, and the effect of inserts in the gene region was more significant than in promoter regions (Fig. 5a). This trend was consistent with LTR-RTs inserted in *R*-genes (Fig. 5b). Gene ontology (GO) analyses indicated that the LTR-RTs-associated genes showed various functions, such as metabolism, cell periphery, response to stress, gene regulation, and system development (Fig. S10). The *Q. lobata* was different from other oaks, mainly enriched in GO regulation of retrotransposon nucleocapsid (GO:0000943), transposition (GO:0032196), and DNA-directed DNA polymerase activity (GO:0003887). KEGG results showed that the LTR-RTs-associated genes were enriched in genetic information processing, transport and catabolism, signal transduction, translation, carbohydrate metabolism, and environmental adaptation (Fig. S11).

**Fig. 6:**
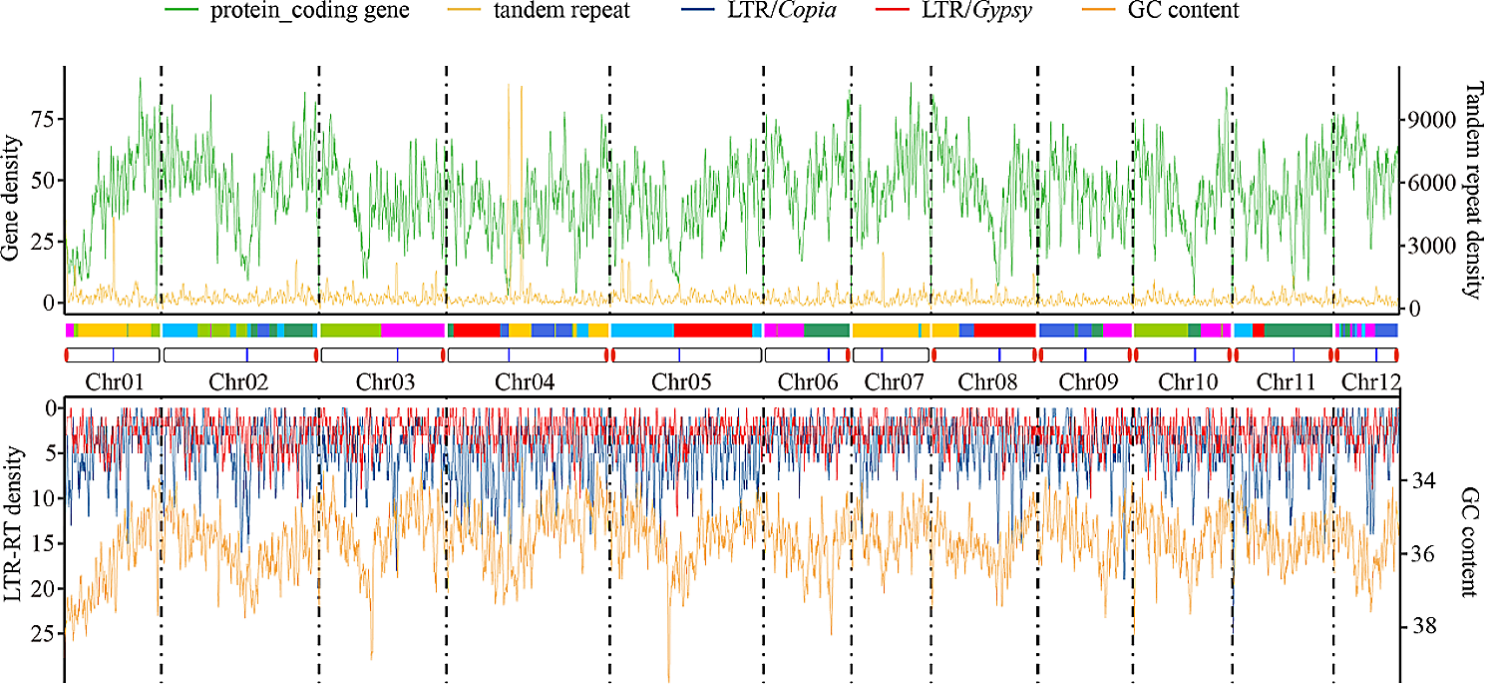
Chromosomal distribution of genes, tandem repeat, LTR-RT density, and GC content. The red dots at both ends of the chromosome indicate the position of the telomere; The blue lines on the chromosome indicate the predicted position of the Centromere region

To investigate the impact of LTR-RTs on chromosomal structure, we analyzed the distribution of genes, tandem repeats, LTR-RTs, and GC content in the *Q. glauca* genome (Fig. 6). The results showed that gene density, LTR-RT density, and GC content had regional special enrichment patterns. For example, the low gene density but high LTR-RT density and GC content were found in the 0-18.2 Mb region of chromosome 1, and chromosomes 4, 9, and 11 have multiple regions with similar characteristics. Chromosomal rearrangement regions in chromosomes 4, 8, and 11 have low gene density and high LTR-RT distribution.

In most chromosomes of *Q. glauca*, there are regions with higher frequencies of LTR/*Copia*, LTR/*Gypsy*, tandem repeats, and GC content, but low-frequency gene density was consistent with the characteristics of the centromere region. Various methods were used to predict the potential centromere regions of *Q. glauca.* First, the enrichment of repeat units along the genome was detected (Fig. S13). A total of six centromere regions were found, and the repeat units varied among chromosomes. 32 bp repeat units were evident in the centromere regions of chromosomes 1, 5, 9, and 11, 79 bp in chromosome 2, and 78 bp in chromosome 4. Second, discontinuous signals in chromatin interaction heat maps were used to predict the potential centromere regions for each chromosome (Fig. S12a). Third, the analysis programs Centromics and the CentroMiner predicted six and twelve potential centromere regions, respectively (Table S5 and Fig. S12b, c). Based on the LTR-RTs distribution and the prediction methods, 12 chromosome centromeres were defined (Fig. 6). The six regions identified by repeat units were highly linked with the centromeres. Eight and six predicted regions correspond to the defined centromeres in the genomic discontinuous signals and CentroMiner results, respectively. In addition, we also used IGV to detect the repeat units in the miss-predicted centromere regions, which were caused by longer repeat units, such as the 367 bp long repeat units in chromosomes 1, 2, 8, and 10, as well as assembly gaps (Fig. S12d).

## Discussion

The ancestor karyotype projection provides evidence for studying the evolutionary history of species by identifying collinear genes and their order [13, 15]. Previous ancestor karyotype projection studies contained undefined regions and only revealed limited karyotype dynamics [11, 12, 50]. This study utilized WGDI to identify the proto-chromosomes by searching for shared intact chromosomes or chromosome-like synteny blocks to complete gap regions [14, 15]. The ancestral karyotype projections of eight oak species from the four sections were established, elucidating the roles of chromosome fusion and arm exchange in the evolution of 12 modern chromosomes. This study could provide new insights into the impact of ancient whole-genome triplication events on karyotype evolution, the role of interspecies chromosome rearrangement in speciation, and the dynamics of oak chromosomal evolution.

As diploids, lineage-specific whole genome duplication events have not occurred in oaks [42]. By completing the gap regions in karyotype projections, the ancestral synteny blocks of all chromosome regions in oak species were defined, which contributes to exploring the differences in common ancestor and species-specific chromosomal evolution of oak species. The interspecific conserved synteny blocks exist between modern oak genomes and ancestral karyotypes from the same ancestor [51]. Previous research used the shared synteny blocks to explore the most intact chromosome as an ancestral proto-chromosome [12, 15]. However, complex rearrangement in oak genomes resulted in the distribution of shared synteny blocks within segments of several chromosomes, making it difficult to precisely explore the common ancestral proto-chromosome. Rearrangement occurs frequently in plant genomes and can promote the evolution of chromosome number, size, structure, and composition [8, 9]. After polyploidization, the following diploidization entails various chromosome rearrangements, such as inversions, translocations, fission and fusion, duplications, and deletions [52]. These events could contribute to the richness of structural diversity of the oak karyotype. Compared to the *Betula pendula* of Betulaceae, the evolution of AEK 6 in the Fagaceae and Betulaceae is relatively conservative, and their chromosomes have undergone complex rearrangements (Fig. S4) [15]. Chromosome rearrangement enriches the chromosomal structural diversity of these two widely distributed and ancient Fagales lineages and contributes to adaptive evolution. This study clarified the evolution of modern *Q. glauca* chromosomes and confirmed the important role of chromosome fusion and arm exchange in karyotype evolution. To elucidate the common ancestor and the specific details of karyotype evolution of oaks, it is necessary to analyze karyotype evolution based on representative genomes of other lineages [13, 15].

Identical and species-specific chromosomal rearrangements within oaks were shown in the ancestor karyotype projection and interspecific synteny relationships. In oaks, research has revealed the importance of natural hybridization and introgression in promoting genetic diversity and the generation of new species [53, 54]. Identical chromosomal rearrangements among oaks are associated with the evolution of *Quercus*’ common ancestor, and these rearrangements may have been preserved in frequent hybridization and have the effect of inhibiting recombination [55, 56]. Species-specific chromosomal variation enriched the lineage-specific diversity of chromosomal structure and contributed to the species reproductive isolation, speciation, and adaptive evolution [3, 9]. The accumulation of chromosomal rearrangements between species is largely incidental to speciation, and affects gene flow and fitness [55, 57]. For example, chromosomal rearrangements may cause postzygotic barriers or suppress the recombination of heterologous karyotypes, which could lead to speciation [58]. Some species-specific chromosome structural variation detected in this study were consistent with previous oak genome research [38–40, 49]. The species-specific inversion and translocation in chromosomes 3 and 5 of *Q. lobata* may be related to the ancient speciation and unique lineage evolution on the west coast of North America. The interspecific chromosome rearrangements appeared irregular among different sections, which could not provide direct evidence for divergence and speciation among oak species. *Q. glauca* and *Q. gliva*, from section *Cyclobalanopsis*, exhibited chromosome inversion in chromosomes 1 and 7, possibly related to speciation and habitat differences. Chromosome rearrangement undoubtedly enriches the diversity of oak karyotypes, and further research on rearrangement sequence should explore interspecific differences, stress resistance, and ecological adaptability in the oak species.

LTR-RTs and polyploidization promote adaptation and shape genomic structure [10]. The proportion of LTR in the oak species varied, ranging from approximately 139.1 Mb (17.2%) in *Q. mongolica* [42] to 371.3 Mb (46.6%) in *Q. variabilis* [43] (Table S1). Previous genomic studies on oaks focused on analyzing LTR-RTs content, with little further identification of intact full-length regions based on different lineages in the *Copia* and *Gypsy* subfamilies. According to conserved protein domains and the REXdb database [59], we identified intact full-length LTR-RTs from 33.8 Mb to 56.8 Mb when excluding some Unknown elements and solo LTRs. The amplification and depletion of LTR-RTs affect genome structure, size, and evolutionary rates [17]. Previous research on Fabaceae and Curcurbitaceae species has shown a significant positive correlation between LTR-RT content and genome size [24, 25]. Similar genome sizes but varying LTR-RTs densities in oaks imply that species-specific evolutionary histories could affected the richness of LTR-RTs across species. Several factors could contribute to the content of LTR-RTs, such as chromosomal rearrangement and solo LTRs content [60–62]. In oaks, *Q. lobata*, with species-specific chromosomal rearrangements, has fewer intact LTR-RTs and solo LTR, which may suggest that the genome maintained relatively stable after speciation. Two species with larger genome sizes, *Q. glauca* and *Q. gilva*, have more intact LTR-RTs and solo LTR, which may suggest rapid evolution in their genomes.

The LTR-RTs are sub-classified into different lineages in oaks, with SIRE and Retand accounting for most of the *Copia* and *Gypsy* subfamilies, respectively. Previous research [24, 25] found the scales and timeframes of activity amplifying LTR-RTs vary dramatically among families, lineages, and species [17]. In oaks, the *Copia*/Ale, *Copia*/SIRE, *Copia*/Angela, and *Gypsy*/Retand lineages exhibited varying amplification and evolutionary patterns. The amplification of different LTR-RTs lineages in the oak genome was a source of intraspecific polymorphism, which is considered an important factor affecting genomic diversity and adaptive evolution [63]. Although both *Q. gilva* and *Q. glauca* belong to the section *Cyclobalanopsis*, *Q. gilva* has more ancient amplification among the four lineages while *Q. glauca* shows recent independent amplification in *Copia*/Angela. Two species of section *Cerris* (*Q. acutissima* and *Q*. *variabilis*) showed more recent amplification in *Gypsy*. The different amplification/loss rates of LTR-RT specific lineages in oak species may imply a difference in the evolutionary rate of the sections and species [17].

Insertion of LTR-RTs into genomes impacts gene expression, regulation, and function, such as changing gene structure or the functional elements in the promoter region [25, 64–66]. Comparative transcriptomic analyses confirmed the suppression function of LTR-RTs inserted in *Q. glauca* genes, consistent with previous studies in Curcurbitaceae and Fabaceae species [24, 25]. In GO enrichment analysis, LTR-RT-associated genes in oaks were enriched in envelope and heterochromatin formation, which were related to SIRE and Retand amplification [67–69]. Meanwhile, the mutations caused by LTR-RT insertion may also affect phenotypes. For example, an LTR-RT inserted into the apple *MdMYB1* gene will increase anthocyanidin accumulation and form red skin [70]. The LTR-RTs insertion in *BoCYP704B1* is the primary cause of the male sterility in cabbage [71]. Therefore, the impact of inserted LTR-RT on gene expression regulation in oak genomes warrants further study.

Through integration and subsequent deletions, LTR-RTs are thought to facilitate subtle restructuring of chromosomal landscapes [9]. LTR/*Copia* and LTR/*Gypsy* were usually mixed with tandem repeats and enriched in plant centromere regions [60, 72, 73]. The pattern of 32, 78, and 79 bp repeat units are highly linked with the centromere regions of six chromosomes in *Q. glauca*, but *Q. lobata* has a consistent repeat unit (148 bp) for each centromere [49]. This result indicated that although the centromeres are conserved function across species, there is diversity in their structure and sequence [74]. The centromere region’s complex and highly repetitive structure often leads to collapse and truncation during genome assembly, which may mean we have not identified all centromeres [75]. During polyploidization and subsequent restoration to diploid, the centromere plays an important role in karyotype rearrangement and speciation [60, 76]. Some chromosomal rearrangement regions in *Q. glauca* exhibited unique patterns of LTR-RTs enrichment. The centromere tandem repeat units were also common in non-centromeres regions in the *Q. glauca* genome, which may be related to the centromere’s loss and formation after chromosome fusion and fission. However, whether ancient centromere repeats still exist in the modern genome and have special functions to maintain the stability of chromosomes remains a mystery [77]. Recent studies have proposed a new genome assembly method that can assemble a highly continuous and completely gap-free reference genome, allowing better identification of all centromere regions and exploring centromere evolution [78, 79]. This study can provide conditions for precise identification of the centromere regions in the oak genome to explore the variation between oaks and its impact on karyotype evolution.

## Conclusions

This study revealed the effects of polyploidization and LTR-RTs amplification on oak genome structure, function, and evolution. We confirmed that after the ancient triplication event from AEK, the oak genomes decreased by nine chromosomes through fusion, fission, and rearrangement, reaching a stable state with 12 chromosomes in modern genomes. After speciation, recent LTR-RTs amplification in different lineages affected their composition and abundance variably in oak species. The insertion of LTR-RTs into genes partly suppresses gene expression. The distribution pattern of LTR-RTs combined with gene density, tandem repeat density, and GC content were used to identify centromere regions in the *Q. glauca* genome. However, in the long evolutionary history of oak species, clarifying the impact of ancestral karyotype evolution and LTR-RTs on genome amplification and chromosomal structural variation needs further verification using more high-quality genomes from related species.

## Methods

### Whole-genome duplication analyses and karyotype projection

Eight oak genomes were obtained from previous literature (Table S1), including two species, *Q. gilva* [39] and *Q. glauca* [40], from section *Cyclobalanopsis*; two species, *Q. acutissima* [37] and *Q*. *variabilis* [43], from section *Cerris*; four species, *Q*. *dentata* [38], *Q*. *lobata* [41], *Q*. *mongolica* [42], and *Q*. *robur* [11], from section *Quercus*. Chromosome sizes ranged from 750 Mb (*Q. acutissima*) to 893 Mb (*Q. dentata*) and gene numbers ranged from 30,820 (*Q. acutissima*) to 39,023 (*Q. glauca*; Table S1).

The projections of the ancestral eudicots karyotype (AEK) and ancestral core eudicots karyotype (ACEK) were reconstructed using WGDI v0.6.5 [14]. First, the protein sequences of the eight oak species were compared with the AEK and ACEK using BLAST v2.12.0 [80] with “-outfmt 6 -evalue 1e-5 -num_alignments 20” parameters. The script generate_conf.py (https://github.com/xuzhougeng/myscripts/blob/master/comparative/generate_conf.py) was used to obtain the gene location and chromosome information required by WGDI. Second, the “-icl” parameter in WGDI was used to identify collinear genes between the modern genomes and the two ancestral karyotypes, and “-bi -c -bk” parameters were used to integrate, filter, and check the synteny blocks. WGDI with the “-km” parameter was used to obtain the mapping results from AEK and ACEK to the oaks karyotype. Finally, homologous dot-plots between the modern genomes and the two ancestral karyotypes were plotted using WGDI, and the ancestral karyotype projections were visualized. The protein sequences of *Q. glauca*, the most complete genome among oak species so far, were compared with those of the other oak genomes using BLAST v2.12.0 [80] to identify the diversity in karyotype evolution and chromosomal rearrangement. Homologous dot-plots between *Q. glauca* and those of other oak species were plotted with the ACEK karyotype mapping results. CD-HIT [81] was used to remove redundant protein sequences with “-c 0.8 -aS 0.8 -d 0” parameters for further constructing phylogenetic trees. Then, OrthoFinder v2.5.4 [82] was used to identify orthologs and construct a maximum likelihood (ML) phylogenetic tree with the “-S diamond -M msa” parameters. We used “-M msa” for multiple sequence alignments (MSA) and used default parameters in MAFFT v7.515 [83] and FastTree v2.1.11 [84] to infer maximum likelihood trees.

### LTR-RTs identification and annotation

We used EDTA v1.9.6 [85] (Extensive de-novo TE Annotator), a comprehensive process tool that integrates the results of several current LTR prediction tools, such as LTR_FINDER [86], LTRharvest [87], and LTR_retriever [88], to build a highly reliable non-redundant TE database, and annotated repeated sequences with RepeatMasker [89]. We used EDTA.pl with the “-species others -step all -anno 1 -sensitive 1” parameters to obtain the TE database for each oak genome. The protein domains of the elements belonging to different lineages of *Copia* or *Gypsy* superfamilies were analyzed using REXdb [27], which was implemented using TEsorter v1.2.5.2 [59]. The recombination caused by the disappearance of internal components will lead to the removal of intact LTR-RTs and the formation of solo LTRs [61, 62]. We extracted solo LTRs from the annotation file generated by the RepeatMasker in EDTA.

To explore LTR-RTs amplification and the disparity in evolution among oak species, we used the formula T = (1 - identity) / 2µ to calculate the transposition time of LTR-RTs, where identity represents the sequence similarity between 5’ and 3’ LTRs obtained from the EDTA analysis, *µ* represents the base substitution rate. The substitution rate 1.01 × 10^− 8^ of *Q. lobate* [49] is the oak substitution rate in this study. To investigate the historical dynamics of different lineages of *Copia* and *Gypsy*, we extracted RT protein domain sequences of diverse lineages in these superfamilies by the concatenate_domains.py script in TEsorter [59]. After sequence alignments were carried out using MAFFT v7.515 [83], ML phylogenetic trees were constructed and visualized using FastTree v2.1.11 [84] and iTOL [90], respectively.

### LTR-RTs associated with genes

We analyzed the number and function of genes that overlap with LTR-RTs. The LTR-RTs overlapping with gene and promoter regions were calculated using the “intersect” function from BEDtools v2.30.0 [91]. Protein sequences of the gene and promoter regions overlapping with LTR-RTs were extracted. GO enrichment analysis of extracted genes was carried out using the eggNOG-mapper [92] online tool and the R package ClusterProfiler [93]. The metabolic pathways were annotated with KAAS [94] and visualized with R package ggplot2 [95].

We used transcriptome data from the leaf, inflorescence, and stem of *Q. glauca* from the NCBI SRA database (BioProject: PRJNA868092) to evaluate the impact of LTR-RTs on the expression of adjacent genes. Hisat2 v2.2.1 [96], Samtools v1.13 [97], and StringTie v2.2.1 [98] were used to compare transcriptome data to the reference genome, sort and index sam files, and obtain the read count. Gene expression level was quantified in TPM (transcripts per million). Paralogous genes were detected using BLAST v2.12.0 [80]. Expression levels of paralogous genes with and without overlapping LTR-RT were compared. We further analyzed the impact of LTR-RTs insertion on the expression level of resistance genes (*R*-genes), as the evolution of *R*-genes is widely considered to be affected by LTR-RT insertion.

### LTR-RTs distribution

LTR/*Copia* and LTR/*Gypsy* were usually mixed with tandem repeats and enriched in plant centromere regions. Combined with previous research [79, 99], we used *Q. glauca* as a reference to scan the regions with a higher frequency of tandem repeat, LTR/*Copia*, and LTR/*Gypsy* distribution and also a higher GC content but low-frequency gene density. The densities of genes, tandem repeats, LTR/*Copia*, and LTR/*Gypsy* were calculated using BEDtools v2.30.0 [91] with parameters “-w 1000000 -s 200000” to make interval “windows” and “-counts -F 0.5” to compute the coverage. The GC content of the *Q. glauca* genome was calculated by seqkit [100] tools with the same sliding window size. The R scripts completed data visualization.

To predict potential centromere regions, we first used the Telomeres_and_Centromeres [99] method to detect the tandem repeats (TRs) by TRF v4.09.1 [101] software with the “2 7 7 80 10 50 500 -f -d -m” parameters, and TRF2GFF (https://github.com/Adamtaranto/TRF2GFF) was used to merge the annotated results. Then we screened high-frequency repeat units in each chromosome, using IGV v2.16.1 [102] to visualize the density of genome annotation, LTR-RTs, and repeat units. Potential centromere regions showed low-frequency peaks of genome and TE and high-frequency peaks of repeat units in IGV. Second, Juicebox v1.11.08 [103] was used to observe the Hi-C heat map of the *Q. glauca* [40] genome. Third, Centromics (https://github.com/zhangrengang/Centromics) and the CentroMiner tools of quarTeT v1.1.1 [78] were used default parameters to predict the potential centromere regions.

### Electronic supplementary material

Below is the link to the electronic supplementary material.

Supplementary Material 1

## Data Availability

The genomes of *Q. gilva*, *Q. lobata*, *Q. mongolica*, and *Q. robur* are available in the NCBI repository (https://www.ncbi.nlm.nih.gov/) with GenBank accession numbers GCA_023736055.1, GCA_001633185.5, GCA_011696235.1, and GCF_932294415.1, respectively. The genomes of *Q. acutissima*, *Q. dentata*, and *Q. glauca* are available in the NGDC repository (https://ngdc.cncb.ac.cn/) with accession numbers GWHBGBO00000000, GWHBRAD00000000, and GWHCAYJ00000000, respectively. The *Q. variabilis* genome is available in the CNGB repository (https://db.cngb.org/) with accession number CNA0051893.

## References

[1] Wu F, Tanksley SD (2010). Chromosomal evolution in the plant family Solanaceae. BMC Genomics.

[2] Soltis DE, Soltis PS (1999). Polyploidy: recurrent formation and genome evolution. Trends Ecol Evol.

[3] Schubert I (2007). Chromosome evolution. Curr Opin Plant Biol.

[4] Jiao Y, Wickett NJ, Ayyampalayam S, Chanderbali AS, Landherr L, Ralph PE, Tomsho LP, Hu Y, Liang H, Soltis PS (2011). Ancestral polyploidy in seed plants and angiosperms. Nature.

[5] Wu S, Han B, Jiao Y (2020). Genetic contribution of paleopolyploidy to adaptive evolution in angiosperms. Mol Plant.

[6] Chanderbali AS, Jin L, Xu Q, Zhang Y, Zhang J, Jian S, Carroll E, Sankoff D, Albert VA, Howarth DG (2022). *Buxus* and *Tetracentron* genomes help resolve eudicot genome history. Nat Commun.

[7] Jiao Y, Leebens-Mack J, Ayyampalayam S, Bowers JE, McKain MR, McNeal J, Rolf M, Ruzicka DR, Wafula E, Wickett NJ (2012). A genome triplication associated with early diversification of the core eudicots. Genome Biol.

[8] Schubert I, Lysak MA (2011). Interpretation of karyotype evolution should consider chromosome structural constraints. Trends Genet.

[9] Eichler EE, Sankoff D (2003). Structural dynamics of eukaryotic chromosome evolution. Science.

[10] Bennetzen JL, Wang H (2014). The contributions of transposable elements to the structure, function, and evolution of plant genomes. Annu Rev Plant Biol.

[11] Plomion C, Aury J-M, Amselem J, Leroy T, Murat F, Duplessis S, Faye S, Francillonne N, Labadie K, Provost GL (2018). Oak genome reveals facets of long lifespan. Nat Plants.

[12] Xie D, Xu Y, Wang J, Liu W, Zhou Q, Luo S, Huang W, He X, Li Q, Yuan J (2019). The wax gourd genomes offer insights into the genetic diversity and ancestral cucurbit karyotype. Nat Commun.

[13] Murat F, Armero A, Pont C, Klopp C, Salse J (2017). Reconstructing the genome of the most recent common ancestor of flowering plants. Nat Genet.

[14] Sun P, Jiao B, Yang Y, Shan L, Li T, Li X, Xi Z, Wang X, Liu J (2022). WGDI: a user-friendly toolkit for evolutionary analyses of whole-genome duplications and ancestral karyotypes. Mol Plant.

[15] Wang Z, Li Y, Sun P, Zhu M, Wang D, Lu Z, Hu H, Xu R, Zhang J, Ma J (2022). A high-quality *Buxus Austro-Yunnanensis* (Buxales) genome provides new insights into karyotype evolution in early eudicots. BMC Biol.

[16] Gantuz M, Morales A, Bertoldi MV, Ibañez VN, Duarte PF, Marfil CF, Masuelli RW (2022). Hybridization and polyploidization effects on LTR-retrotransposon activation in potato genome. J Plant Res.

[17] Zhao M, Ma J (2013). Co-evolution of plant LTR-retrotransposons and their host genomes. Protein cell.

[18] Baniaga AE, Barker MS (2019). Nuclear genome size is positively correlated with median LTR-RT insertion time in fern and lycophyte genomes. Am Fern J.

[19] Mehrotra S, Goyal V (2014). Repetitive sequences in plant nuclear DNA: types, distribution, evolution and function. Genom Proteom Bioinf.

[20] Biscotti MA, Olmo E, Heslop-Harrison JS (2015). Repetitive DNA in eukaryotic genomes. Chromosome Res.

[21] Baucom RS, Estill JC, Chaparro C, Upshaw N, Jogi A, Deragon J-M, Westerman P, SanMigue PJ, Bennetzen JL (2009). Exceptional diversity, non-random distribution, and rapid evolution of retroelements in the B73 maize genome. PLoS Genet.

[22] Xia E, Tong W, Hou Y, An Y, Chen L, Wu Q, Liu Y, Yu J, Li F, Li R (2020). The reference genome of tea plant and resequencing of 81 diverse accessions provide insights into its genome evolution and adaptation. Mol Plant.

[23] Li G, Wang L, Yang J, He H, Jin H, Li X, Ren T, Ren Z, Li F, Han X (2021). A high-quality genome assembly highlights rye genomic characteristics and agronomically important genes. Nat Genet.

[24] Li S, She H, Yang L, Lan L, Zhang X, Wang L, Zhang Y, Li N, Deng C, Qian W (2022). Impact of LTR-retrotransposons on genome structure, evolution, and function in Curcurbitaceae species. Int J Mol Sci.

[25] Yang L, Zhang X, Wang L, Li Y, Li X, Yang Y, Su Q, Chen N, Zhang Y, Li N (2023). Lineage-specific amplification and epigenetic regulation of LTR-retrotransposons contribute to the structure, evolution, and function of Fabaceae species. BMC Genomics.

[26] Wicker T, Sabot F, Hua-Van A, Bennetzen JL, Capy P, Chalhoub B, Flavell A, Leroy P, Morgante M, Panaud O (2007). A unified classification system for eukaryotic transposable elements. Nat Rev Genet.

[27] Neumann P, Novák P, Hoštáková N, Macas J (2019). Systematic survey of plant LTR-retrotransposons elucidates phylogenetic relationships of their polyprotein domains and provides a reference for element classification. Mob DNA.

[28] Yuan J, Jiang S, Jian J, Liu M, Yue Z, Xu J, Li J, Xu C, Lin L, Jing L (2022). Genomic basis of the giga-chromosomes and giga-genome of tree peony *Paeonia Ostii*. Nat Commun.

[29] Du J, Tian Z, Hans CS, Laten HM, Cannon SB, Jackson SA, Shoemaker RC, Ma J (2010). Evolutionary conservation, diversity and specificity of LTR-retrotransposons in flowering plants: insights from genome‐wide analysis and multi‐specific comparison. Plant J.

[30] De Souza TB, Chaluvadi SR, Johnen L, Marques A, González-Elizondo MS, Bennetzen JL, Vanzela AL (2018). Analysis of retrotransposon abundance, diversity and distribution in holocentric *Eleocharis* (Cyperaceae) genomes. Ann Bot.

[31] Bennetzen JL, Ma J, Devos KM (2005). Mechanisms of recent genome size variation in flowering plants. Ann Bot.

[32] Liu Z, Yue W, Li D, Wang RR-C, Kong X, Lu K, Wang G, Dong Y, Jin W, Zhang X (2008). Structure and dynamics of retrotransposons at wheat centromeres and pericentromeres. Chromosoma.

[33] Kim S, Choi D (2018). New role of LTR-retrotransposons for emergence and expansion of disease-resistance genes and high-copy gene families in plants. BMB Rep.

[34] Bahmani M, Forouzan S, Fazeli-Moghadam E, Rafieian-Kopaei M, Adineh A, Saberianpour S (2015). Oak (*Quercus branti*): an overview. J Chem Pharm Res.

[35] Wang Y, Xu C, Wang Q, Jiang Y, Qin L (2022). Germplasm resources of oaks (*Quercus* L.) in China: utilization and prospects. Biology.

[36] Burlacu E, Nisca A, Tanase C (2020). A comprehensive review of phytochemistry and biological activities of *Quercus* species. Forests.

[37] Fu R, Zhu Y, Liu Y, Feng Y, Lu R-S, Li Y, Li P, Kremer A, Lascoux M, Chen J (2022). Genome-wide analyses of introgression between two sympatric Asian oak species. Nat Ecol Evol.

[38] Wang W, He X, Yan X, Ma B, Lu C, Wu J, Zheng Y, Wang W, Xue W, Tian X (2023). Chromosome-scale genome assembly and insights into the metabolome and gene regulation of leaf color transition in an important oak species, *Quercus dentata*. New Phytol.

[39] Zhou X, Liu N, Jiang X, Qin Z, Farooq TH, Cao F, Li H (2022). A chromosome-scale genome assembly of *Quercus gilva*: insights into the evolution of *Quercus* section *Cyclobalanopsis* (Fagaceae). Front Plant Sci.

[40] Luo C, Li T, Jiang X, Song Y, Fan T, Shen X, Yi R, Ao X, Xu G, Deng M. High-quality haplotype-resolved genome assemblies of ring-cup oak (*Quercus glauca*) provide insight into the demographic dynamics of a dominant tree in East Asia subtropics evergreen broadleaved forests. Mol Ecol Resour. 2023;e13914. 10.1111/1755-0998.13914 38108568

[41] Sork VL, Fitz-Gibbon ST, Puiu D, Crepeau M, Gugger PF, Sherman R, Stevens K, Langley CH, Pellegrini M, Salzberg SL. First draft assembly and annotation of the genome of a California endemic oak *Quercus lobata* Née (Fagaceae). G3: Genes, Genomes, Genet. 2016;6(11):3485-95. 10.1534/g3.116.030411 PMC5100847 27621377

[42] Ai W, Liu Y, Mei M, Zhang X, Tan E, Liu H, Han X, Zhan H, Lu X (2022). A chromosome-scale genome assembly of the Mongolian oak (*Quercus mongolica*). Mol Ecol Resour.

[43] Han B, Wang L, Xian Y, Xie X, Li W, Zhao Y, Zhang R, Qin X, Li D, Jia H (2022). A chromosome-level genome assembly of the Chinese cork oak (*Quercus variabilis*). Front Plant Sci.

[44] Yang Y, Zhou T, Duan D, Yang J, Feng L, Zhao G (2016). Comparative analysis of the complete chloroplast genomes of five *Quercus* species. Front Plant Sci.

[45] Deng M, Jiang XL, Hipp A, Manos P, Hahn M. Phylogeny and biogeography of East Asian evergreen oaks (*Quercus* section *Cyclobalanopsis*; Fagaceae): Insights into the Cenozoic history of evergreen broad-leaved forests in subtropical Asia. Mol Phylogenet Evol. 2018;119: 170–81. 10.1016/j.ympev.2017.11.003 29175095

[46] Yang Y, Zhou T, Qian Z, Zhao G (2021). Phylogenetic relationships in Chinese oaks (Fagaceae, *Quercus*): evidence from plastid genome using low-coverage whole genome sequencing. Genomics.

[47] Hipp AL, Manos PS, Hahn M, Avishai M, Bodénès C, Cavender-Bares J, Crow AA, Deng M, Denk T, Fitz-Gibbon S (2020). Genomic landscape of the global oak phylogeny. New Phytol.

[48] Jiang X, Hipp AL, Deng M, Su T, Zhou Z, Yan M (2019). East Asian origins of European holly oaks (*Quercus* section *Ilex* Loudon) via the Tibet-Himalaya. J Biogeogr.

[49] Sork VL, Cokus SJ, Fitz-Gibbon ST, Zimin AV, Puiu D, Garcia JA, Gugger PF, Henriquez CL, Zhen Y, Lohmueller KE (2022). High-quality genome and methylomes illustrate features underlying evolutionary success of oaks. Nat Commun.

[50] Salse J (2016). Ancestors of modern plant crops. Curr Opin Plant Biol.

[51] Wang Z, Wang J, Pan Y, Lei T, Ge W, Wang L, Zhang L, Li Y, Zhao K, Liu T (2019). Reconstruction of evolutionary trajectories of chromosomes unraveled independent genomic repatterning between Triticeae and *Brachypodium*. BMC Genomics.

[52] Li S, Su T, Cheng G, Wang B, Li X, Deng C, Gao W (2017). Chromosome evolution in connection with repetitive sequences and epigenetics in plants. Genes.

[53] Petit RJ, Bodénès C, Ducousso A, Roussel G, Kremer A (2004). Hybridization as a mechanism of invasion in oaks. New Phytol.

[54] Wei G, Li X, Fang Y (2021). Sympatric genome size variation and hybridization of four oak species as determined by flow cytometry genome size variation and hybridization. Ecol Evol.

[55] Lucek K, Giménez MD, Joron M, Rafajlović M, Searle JB, Walden N, Westram AM, Faria R (2023). The impact of chromosomal rearrangements in speciation: from micro-to macroevolution. Cold Spring Harb Perspect Biol.

[56] Faria R, Navarro A (2010). Chromosomal speciation revisited: rearranging theory with pieces of evidence. Trends Ecol Evol.

[57] Rieseberg LH (2001). Chromosomal rearrangements and speciation. Trends Ecol Evol.

[58] Kirkpatrick M, Barton N (2006). Chromosome inversions, local adaptation and speciation. Genetics.

[59] Zhang R, Li G, Wang X, Dainat J, Wang Z, Ou S, Ma Y (2022). TEsorter: an accurate and fast method to classify LTR-retrotransposons in plant genomes. Hortic Res.

[60] Hofstatter PG, Thangavel G, Lux T, Neumann P, Vondrak T, Novak P, Zhang M, Costa L, Castellani M, Scott A (2022). Repeat-based holocentromeres influence genome architecture and karyotype evolution. Cell.

[61] Vitte C, Panaud O (2003). Formation of solo-LTRs through unequal homologous recombination counterbalances amplifications of LTR retrotransposons in rice *Oryza sativa* L. Mol Biol Evol.

[62] Vitte C, Panaud O (2005). LTR retrotransposons and flowering plant genome size: emergence of the increase/decrease model. Cytogenet Genome Res.

[63] Stritt C, Wyler M, Gimmi EL, Pippel M, Roulin AC (2020). Diversity, dynamics and effects of long terminal repeat retrotransposons in the model grass *Brachypodium distachyon*. New Phytol.

[64] Bui QT, Grandbastien M-A. LTR retrotransposons as controlling elements of genome response to stress? Plant transposable elements: impact on genome structure and function. 2012;24;273– 96.

[65] Zhao Y, Li X, Xie J, Xu W, Chen S, Zhang X, Liu S, Wu J, Kassaby YA, Zhang D (2022). Transposable elements: distribution, polymorphism, and climate adaptation in *Populus*. Front Plant Sci.

[66] Grandbastien M-A (2015). LTR retrotransposons, handy hitchhikers of plant regulation and stress response. BBA-Gene Regul Mech.

[67] Havecker ER, Voytas DF (2003). The soybean retroelement *SIRE*1 uses stop codon suppression to express its envelope-like protein. EMBO Rep.

[68] Laten HM, Majumdar A, Gaucher EA (1998). *SIRE-1*, a *copia/Ty1*-like retroelement from soybean, encodes a retroviral envelope-like protein. Proc Natl Acad Sci.

[69] Kejnovsky E, Kubat Z, Macas J, Hobza R, Mracek J, Vyskot B (2006). *Retand*: a novel family of gypsy-like retrotransposons harboring an amplified tandem repeat. Mol Genet Genomics.

[70] Zhang L, Hu J, Han X, Li J, Gao Y, Richards CM, Zhang C, Tian Y, Liu G, Gul H (2019). A high-quality apple genome assembly reveals the association of a retrotransposon and red fruit colour. Nat Commun.

[71] Ji J, Yang L, Fang Z, Zhuang M, Zhang Y, Lv H, Liu Y, Li Z (2017). Recessive male sterility in cabbage (*Brassica oleracea* var. *capitata*) caused by loss of function of *BoCYP704B1* due to the insertion of a LTR-retrotransposon. Theor Appl Genet.

[72] Guo N, Wang S, Gao L, Liu Y, Wang X, Lai E, Duan M, Wang G, Li J, Yang M (2021). Genome sequencing sheds light on the contribution of structural variants to *Brassica oleracea* diversification. BMC Biol.

[73] Rajarammohan S, Pental D, Kaur J (2019). Near-complete genome assembly of *Alternaria brassicae*—A necrotrophic pathogen of *Brassica* crops. Mol Plant Microbe in.

[74] Balzano E, Giunta S (2020). Centromeres under pressure: evolutionary innovation in conflict with conserved function. Genes.

[75] Tørresen OK, Star B, Mier P, Andrade-Navarro MA, Bateman A, Jarnot P, Gruca A, Grynbery M, Kajava AV, Promponas VJ (2019). Tandem repeats lead to sequence assembly errors and impose multi-level challenges for genome and protein databases. Nucleic Acids Res.

[76] Marshall OJ, Chueh AC, Wong LH, Choo KA (2008). Neocentromeres: new insights into centromere structure, disease development, and karyotype evolution. Am J Hum Genet.

[77] Melters DP, Bradnam KR, Young HA, Telis N, May MR, Ruby JG, Sebra R, Peluso P, Eid J, Rank D (2013). Comparative analysis of tandem repeats from hundreds of species reveals unique insights into centromere evolution. Genome Biol.

[78] Lin Y, Ye C, Li X, Chen Q, Wu Y, Zhang F, Pan R, Zhang S, Chen S, Wang X (2023). quarTeT: a telomere-to-telomere toolkit for gap-free genome assembly and centromeric repeat identification. Hortic Res.

[79] Yue J, Chen Q, Wang Y, Zhang L, Ye C, Wang X, Cao S, Lin Y, Huang W, Xian H (2023). Telomere-to-telomere and gap-free reference genome assembly of the kiwifruit *Actinidia chinensis*. Hortic Res.

[80] Mount DW (2007). Using the basic local alignment search tool (BLAST). Cold Spring Harb Protoc.

[81] Li W, Godzik A (2006). Cd-hit: a fast program for clustering and comparing large sets of protein or nucleotide sequences. Bioinformatics.

[82] Emms DM, Kelly S (2019). OrthoFinder: phylogenetic orthology inference for comparative genomics. Genome Biol.

[83] Katoh K, Standley DM (2013). MAFFT multiple sequence alignment software version 7: improvements in performance and usability. Mol Biol Evol.

[84] Price MN, Dehal PS, Arkin AP (2010). FastTree 2–approximately maximum-likelihood trees for large alignments. PLoS ONE.

[85] Ou S, Su W, Liao Y, Chougule K, Agda JR, Hellinga AJ, Blanco Lugo CS, Elliott TA, Ware D, Peterson T (2019). Benchmarking transposable element annotation methods for creation of a streamlined, comprehensive pipeline. Genome Biol.

[86] Xu Z, Wang H (2007). LTR_FINDER: an efficient tool for the prediction of full-length LTR retrotransposons. Nucleic Acids Res.

[87] Ellinghaus D, Kurtz S, Willhoeft U (2008). LTRharvest, an efficient and flexible software for de novo detection of LTR retrotransposons. BMC Bioinformatics.

[88] Ou S, Jiang N (2018). LTR_retriever: a highly accurate and sensitive program for identification of long terminal repeat retrotransposons. Plant Physiol.

[89] Chen N (2004). Using repeat Masker to identify repetitive elements in genomic sequences. Curr Protoc Bioinf.

[90] Letunic I, Bork P (2021). Interactive tree of life (iTOL) v5: an online tool for phylogenetic tree display and annotation. Nucleic Acids Res.

[91] Quinlan AR, Hall IM (2010). BEDTools: a flexible suite of utilities for comparing genomic features. Bioinformatics.

[92] Cantalapiedra CP, Hernández-Plaza A, Letunic I, Bork P, Huerta-Cepas J (2021). eggNOG-mapper v2: functional annotation, orthology assignments, and domain prediction at the metagenomic scale. Mol Biol Evol.

[93] Yu G, Wang L, Han Y, He Q (2012). clusterProfiler: an R package for comparing biological themes among gene clusters. Omics.

[94] Moriya Y, Itoh M, Okuda S, Yoshizawa AC, Kanehisa M (2007). KAAS: an automatic genome annotation and pathway reconstruction server. Nucleic Acids Res.

[95] Wickham H (2011). ggplot2. WIREs Comp Stat.

[96] Kim D, Paggi JM, Park C, Bennett C, Salzberg SL (2019). Graph-based genome alignment and genotyping with HISAT2 and HISAT-genotype. Nat Biotechnol.

[97] Li H, Handsaker B, Wysoker A, Fennell T, Ruan J, Homer N (2009). The sequence alignment/map format and SAMtools. Bioinformatics.

[98] Pertea M, Pertea GM, Antonescu CM, Chang T-C, Mendell JT, Salzberg SL (2015). StringTie enables improved reconstruction of a transcriptome from RNA-seq reads. Nat Biotechnol.

[99] Shi X, Cao S, Wang X, Huang S, Wang Y, Liu Z, Liu W, Leng X, Peng Y, Wang N (2023). The complete reference genome for grapevine (*Vitis vinifera* L.) genetics and breeding. Hortic Res.

[100] Shen W, Le S, Li Y, Hu F (2016). SeqKit: a cross-platform and ultrafast toolkit for FASTA/Q file manipulation. PLoS ONE.

[101] Benson G (1999). Tandem repeats finder: a program to analyze DNA sequences. Nucleic Acids Res.

[102] Thorvaldsdóttir H, Robinson JT, Mesirov JP (2013). Integrative Genomics Viewer (IGV): high-performance genomics data visualization and exploration. Brief Bioinform.

[103] Durand NC, Robinson JT, Shamim MS, Machol I, Mesirov JP, Lander ES, Aiden EL (2016). Juicebox provides a visualization system for Hi-C contact maps with unlimited zoom. Cell Syst.

