# Supplementary material for: Karyotype and LTR-RTs analysis provide insights into oak genomic evolution
Source: BMC Genomics. 2024 Apr 3;25:328. doi: 10.1186/s12864-024-10177-6 (PMC10988972; doi:10.1186/s12864-024-10177-6)
Supplement: Supplementary file 1 — Supplementary Material 1 [file 12864_2024_10177_MOESM1_ESM.docx]

The following Supporting Information is available for this article:

**Supplemental Table 1.** Genomic information of oak species.

**Supplemental Table 2.** Genomic collinearity of AEK, ACEK, and oak species.

**Supplemental Table 3.** Detection and characteristics of full-length LTR-RTs in oak species.

**Supplemental Table 4.** Number of LTR-RTs inserted within gene and promoter regions.

**Supplemental Table 5.** Location information of centromere regions predicted by Centromics and quarTeT methods in *Q. glauca*.

**Supplemental Fig. 1** Homologous gene dot-plots between ancestral AEK and eight oak species.

**Supplemental Fig. 2** Homologous gene dot-plots between ancestral ACEK and eight oak species.

**Supplemental Fig. 3** Karyotype (ACEK) projections for eight oak species.

**Supplemental Fig. 4** Homologous gene dot-plot between *Q. glauca* and *Betula pendula*.

**Supplemental Fig. 5** Homologous gene dot-plots between *Q. glauca* and itself and seven other oak species.

**Supplemental Fig. 6** Sequence identity distribution of LTR-RTs hits represented in a swarm plot for eight oak species. **(a)** Identity distribution of all elements; **(b)** Identity distribution of all *Copia*; **(c)** Identity distribution of all *Gypsy*.

**Supplemental Fig. 7** Number of full-length LTR-RTs belonging to different lineages of *Copia* and *Gypsy* superfamilies.

**Supplemental Fig. 8** Insertion time of the amplified LTR-RTs lineages in oak species (MYA, millions of years ago). **(a)** Insertion time of *Copia*/Ale. **(b)** Insertion time of *Copia*/SIRE; **(c)** Insertion time of *Copia*/Angela in *Q. acutissima,* *Q. gilva*, *Q. glauca*, and *Q. variabilis*; **(d)** Insertion time of *Gypsy*/Retand in all oak species.

**Supplemental Fig. 9** Number of LTR-RTs inserted within gene and promoter. **(a)** Number of full-length LTR-RTs belonging to *Copia* and *Gypsy* superfamilies; **(b)** Number of different lineages of *Copia* superfamily; **(d)** Number of different lineages of *Gypsy* superfamily.

**Supplemental Fig. 10** GO enrichment analysis of LTR-RT-associated genes among eight oak species. **(a)** GO enrichment of LTR-RT-associated genes with LTR-RTs insertion in genes; **(b)** GO enrichment of LTR-RT-associated genes with LTR-RTs insertion in promoter regions.

**Supplemental Fig. 11** KEGG enrichment analysis of LTR-RT-associated genes among eight oak species. **(a)** KEGG enrichment of LTR-RT-associated genes with LTR-RTs insertion in genes; **(b)** KEGG enrichment of LTR-RT-associated genes with LTR-RTs insertion in promoter regions.

**Supplemental Fig. 12** Map of centromeres location information predicted by various methods. **(a)** Discontinuous signals in the chromatin interaction heat maps; **(b)** Centromere localization detection results of method Centromics; **(c)** Centromere localization detection results of CentroMiner tools of quarTeT; **(d)** High-frequency repeat units of Telomeres_and_Centromeres results.

**Supplemental Fig. 13** Repeat annotation in *Q. glauca* reference genome.

**Supplemental Table 1.** Genomic information of oak species.

| Species | Assembly level | Genome size (Mb) | Chromosome size (Mb) | Total genes | TEs content (Mb) | TE ratio (%) | LTR content (Mb) |
| --- | --- | --- | --- | --- | --- | --- | --- |
| *Quercus* *acutissima* | chromosome | 758 | 750 | 30820 | 546.7 | 57.1 | 220.7 |
| *Quercus dentata* | chromosome | 893 | 893 | 31584 | 478.7 | 53.6 | 309.2 |
| *Quercus gilva* | chromosome | 890 | 859 | 36442 | 512.3 | 57.6 | 166.2 |
| *Quercus glauca* | chromosome | 903 | 865 | 39023 | 668.3 | 77.3 | 168.7 |
| *Quercus lobata* | chromosome | 845 | 811 | 36703 | 447.3 | 52.0 | 179.0 |
| *Quercus mongolica* | chromosome | 810 | 775 | 33489 | 419.4 | 51.8 | 139.1 |
| *Quercus robur* | chromosome | 790 | 717 | 32231 | 372.6 | 52.0 | 197.5 |
| *Quercus variabilis* | chromosome | 796 | 787 | 32466 | 486.5 | 61.1 | 371.3 |

**Supplemental Table 2.** Genomic collinearity of AEK, ACEK, and oak species.

| Species | Genomic collinearity to AEK | | Genomic collinearity to ACEK | | Genomic collinearity to Q. glauca | | | |
| --- | --- | --- | --- | --- | --- | --- | --- | --- |
|  | Blocks number | Gene pairs number | Blocks number | Gene pairs number | Blocks number | Gene pairs number | Orthologous blocks/gene pairs | Paralogous blocks/gene pairs |
| *Q. acutissima* | 332 | 6808 | 821 | 18440 | 1072 | 20659 | 734/17355 | 338/3344 |
| *Q. dentata* | 416 | 7295 | 974 | 19041 | 1116 | 21099 | 794/17870 | 322/3229 |
| *Q. gilva* | 421 | 6103 | 1018 | 15865 | 1427 | 23531 | 1104/20656 | 323/2875 |
| *Q. glauca* | 405 | 6250 | 1045 | 16393 | 1114 | 13553 | 785/10382 | 329/3171 |
| *Q. lobata* | 505 | 15865 | 1107 | 20446 | 1338 | 23403 | 939/19773 | 399/3630 |
| *Q. mongolica* | 369 | 7321 | 924 | 19775 | 1144 | 21869 | 805/18525 | 339/3344 |
| *Q. robur* | 430 | 7669 | 964 | 19923 | 1162 | 22730 | 815/19254 | 347/3476 |
| *Q. variabilis* | 306 | 5929 | 782 | 16154 | 907 | 19027 | 597/16131 | 310/2896 |

**Supplemental Table 3.** Detection and characteristics of full-length LTR-RTs in oak species.

| Species | Total chromosome length (Mb) | Full-length LTR-RTs | | | Solo LTR | | Number of each type | | | Average length of each type (bp) | | |
| --- | --- | --- | --- | --- | --- | --- | --- | --- | --- | --- | --- | --- |
|  |  | Number | Cumulative length (Mb) | Number/Mb | Number | Cumulative length (Mb) | *Copia* | *Gypsy* | Unknown | *Copia* | *Gypsy* | Unknown |
| *Q. acutissima* | 750 | 4791 | 37.83 | 6.4 | 146298 | 128.46 | 2344 | 2019 | 428 | 7069 | 9430 | 5142 |
| *Q. dentata* | 893 | 4102 | 33.77 | 4.6 | 152408 | 136.69 | 2381 | 1091 | 630 | 7776 | 10556 | 5936 |
| *Q. gilva* | 859 | 6879 | 56.79 | 8.0 | 101209 | 122.86 | 3642 | 2423 | 814 | 7699 | 9872 | 5898 |
| *Q. gluaca* | 865 | 7455 | 55.55 | 8.6 | 118688 | 134.59 | 3952 | 2541 | 962 | 7600 | 10029 | 5776 |
| *Q. lobata* | 811 | 4461 | 35.89 | 5.5 | 88713 | 106.47 | 2050 | 1772 | 639 | 7278 | 9900 | 5335 |
| *Q. mongolica* | 775 | 5618 | 47.03 | 7.3 | 86029 | 93.22 | 2751 | 2164 | 703 | 7720 | 10092 | 5576 |
| *Q. robur* | 717 | 5595 | 47.25 | 7.8 | 83118 | 94.48 | 2795 | 2171 | 629 | 7666 | 10158 | 5930 |
| *Q. variabilis* | 787 | 5248 | 41.99 | 6.7 | 84415 | 129.80 | 2664 | 2163 | 421 | 7314 | 9395 | 5178 |

**Supplemental Table 4.** Number of LTR-RTs inserted within gene and promoter regions.

| Species | Number of LTR-RTs inserted within gene | | | | Number of LTR-RTs inserted within promoter | | | | Number of LTR-RTs inserted within R-genes | Number of LTR-RTs inserted within R-genes promoter regions |
| --- | --- | --- | --- | --- | --- | --- | --- | --- | --- | --- |
|  | Total | *Copia* | *Gypsy* | Unknown | Total | *Copia* | *Gypsy* | Unknown |  |  |
| *Q. acutissima* | 501 | 272 | 195 | 34 | 1035 | 400 | 563 | 72 | 33 | 55 |
| *Q. dentata* | 350 | 209 | 106 | 35 | 745 | 372 | 298 | 75 | 22 | 63 |
| *Q. gilva* | 302 | 174 | 101 | 27 | 1443 | 615 | 705 | 123 | 10 | 33 |
| *Q. glauca* | 437 | 263 | 121 | 53 | 1772 | 804 | 787 | 181 | 13 | 36 |
| *Q. lobata* | 914 | 243 | 626 | 45 | 293 | 81 | 179 | 33 | 80 | 23 |
| *Q. mongolica* | 1267 | 513 | 635 | 119 | 1531 | 542 | 867 | 122 | 54 | 66 |
| *Q. robur* | 1495 | 551 | 851 | 93 | 1760 | 633 | 1024 | 103 | 86 | 52 |
| *Q. variabilis* | 550 | 282 | 238 | 30 | 1057 | 451 | 545 | 61 | 48 | 64 |

**Supplemental Table 5.** Location information of centromere regions predicted by Centromics and quarTeT methods in *Q. glauca*.

| Chromosome | Centromics | | quarTeT | |
| --- | --- | --- | --- | --- |
|  | Start site (bp) | End site (bp) | Start site (bp) | End site (bp) |
| Chr01 | 59640000 | 60570000 | 31452150 | 31839403 |
| Chr02 | 101110000 | 101500000 | 86814905 | 87194684 |
| Chr03 |  |  | 4664143 | 4865439 |
| Chr04 |  |  | 46734945 | 49137326 |
| Chr05 | 37190000 | 37320000 | 11526862 | 13049109 |
| Chr06 | 53690000 | 54180000 | 47526203 | 47628736 |
| Chr07 |  |  | 20376565 | 21408450 |
| Chr08 | 1 | 1160000 | 1851224 | 1977564 |
| Chr09 |  |  | 13565850 | 13737604 |
| Chr10 | 1 | 1250000 | 1 | 1231101 |
| Chr11 |  |  | 32251787 | 32369422 |
| Chr12 |  |  | 27216531 | 27320280 |


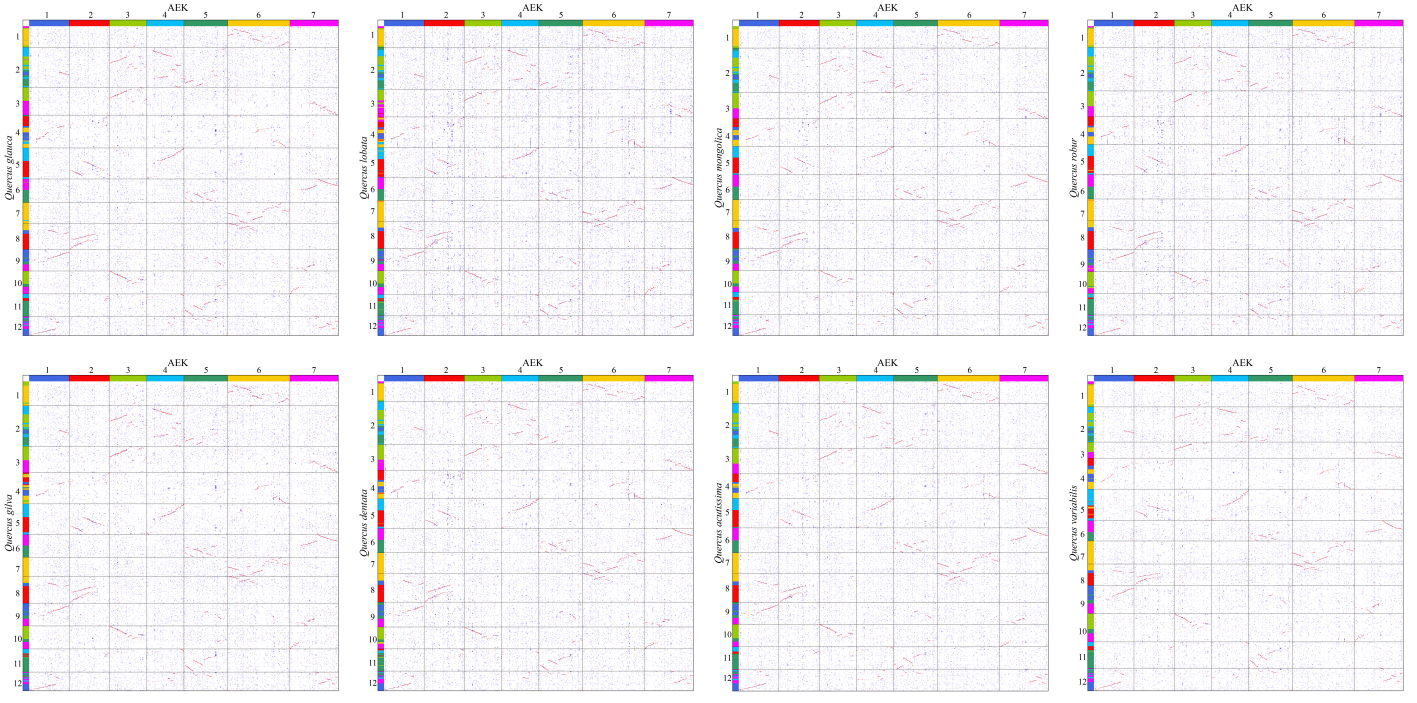


**Supplemental Fig. 1** Homologous gene dot-plots between ancestral AEK and eight oak species.


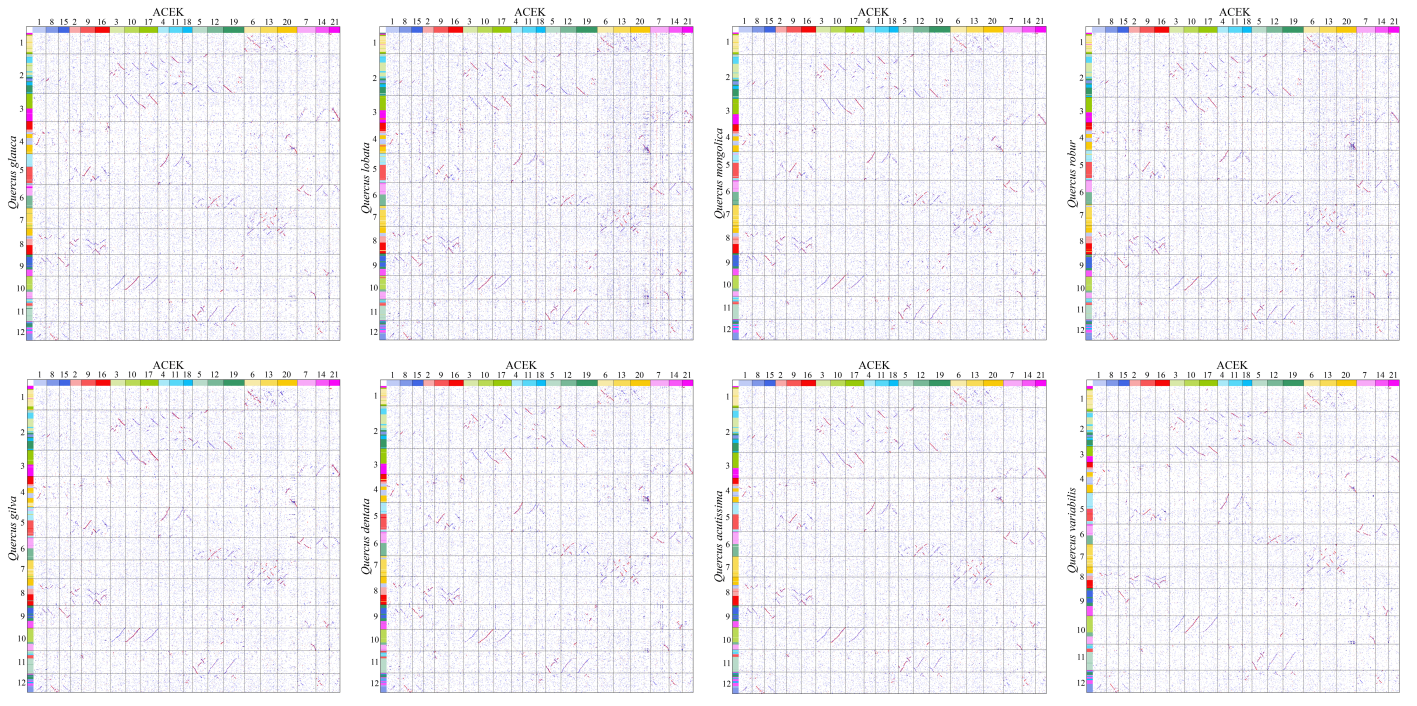


**Supplemental Fig. 2** Homologous gene dot-plots between ACEK and eight oak species.


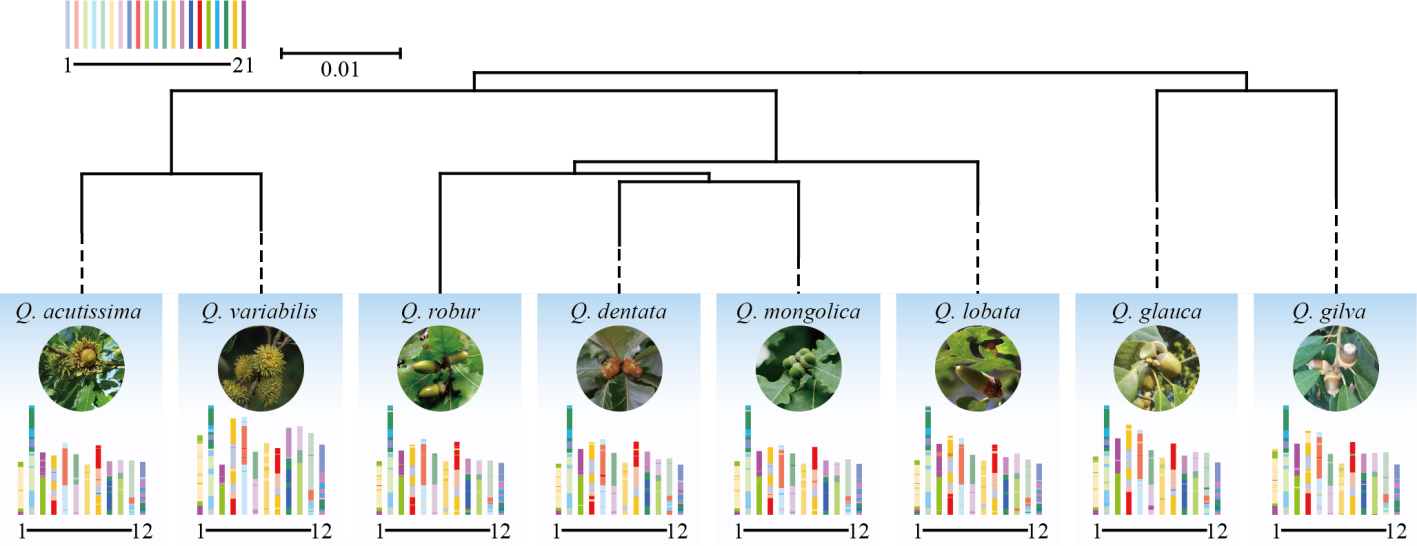


**Supplemental Fig. 3** Karyotype (ACEK) projections for eight oak species.

**
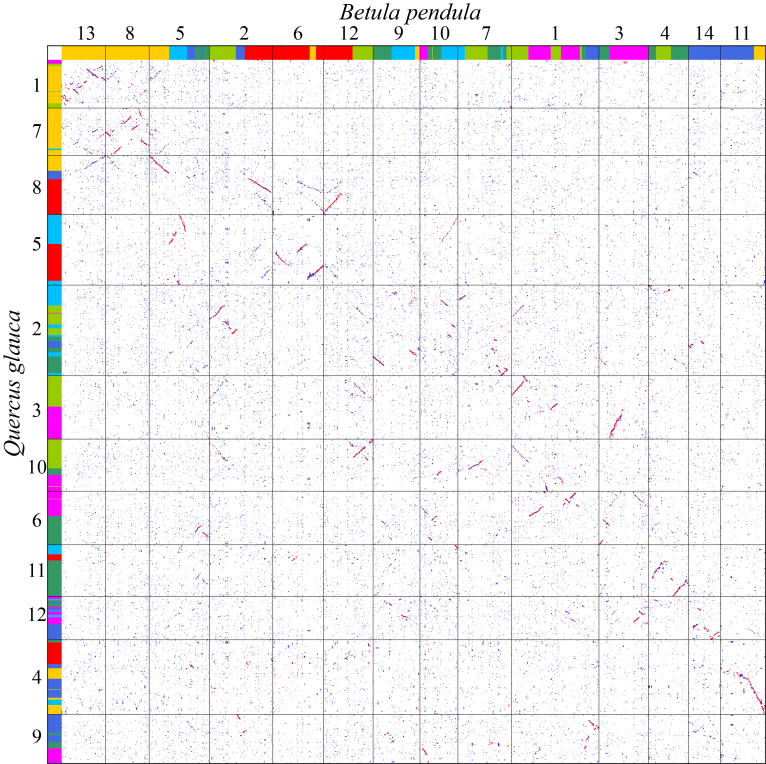
**

**Supplemental Fig. 4** Homologous gene dot-plot between *Q. glauca* and *B. pendula*.


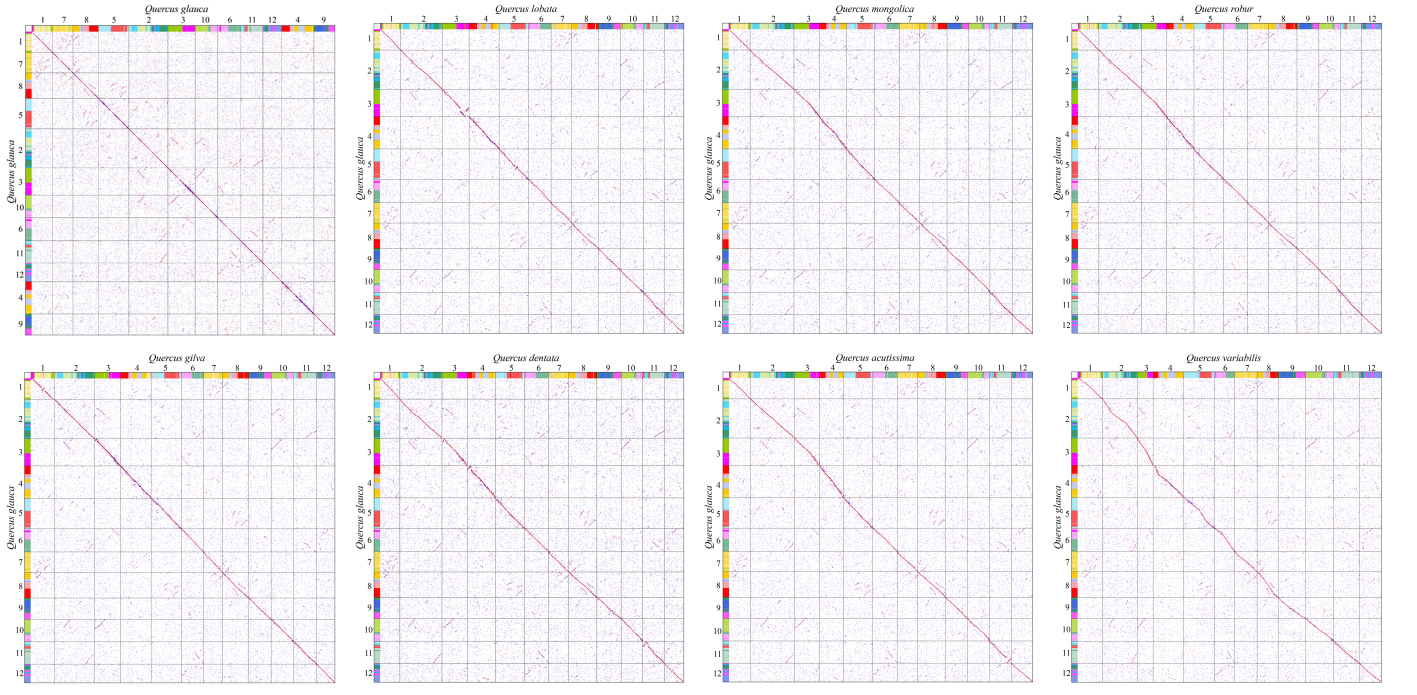


**Supplemental Fig. 5** Homologous gene dot-plots between *Q. glauca* and itself and seven other oak species.


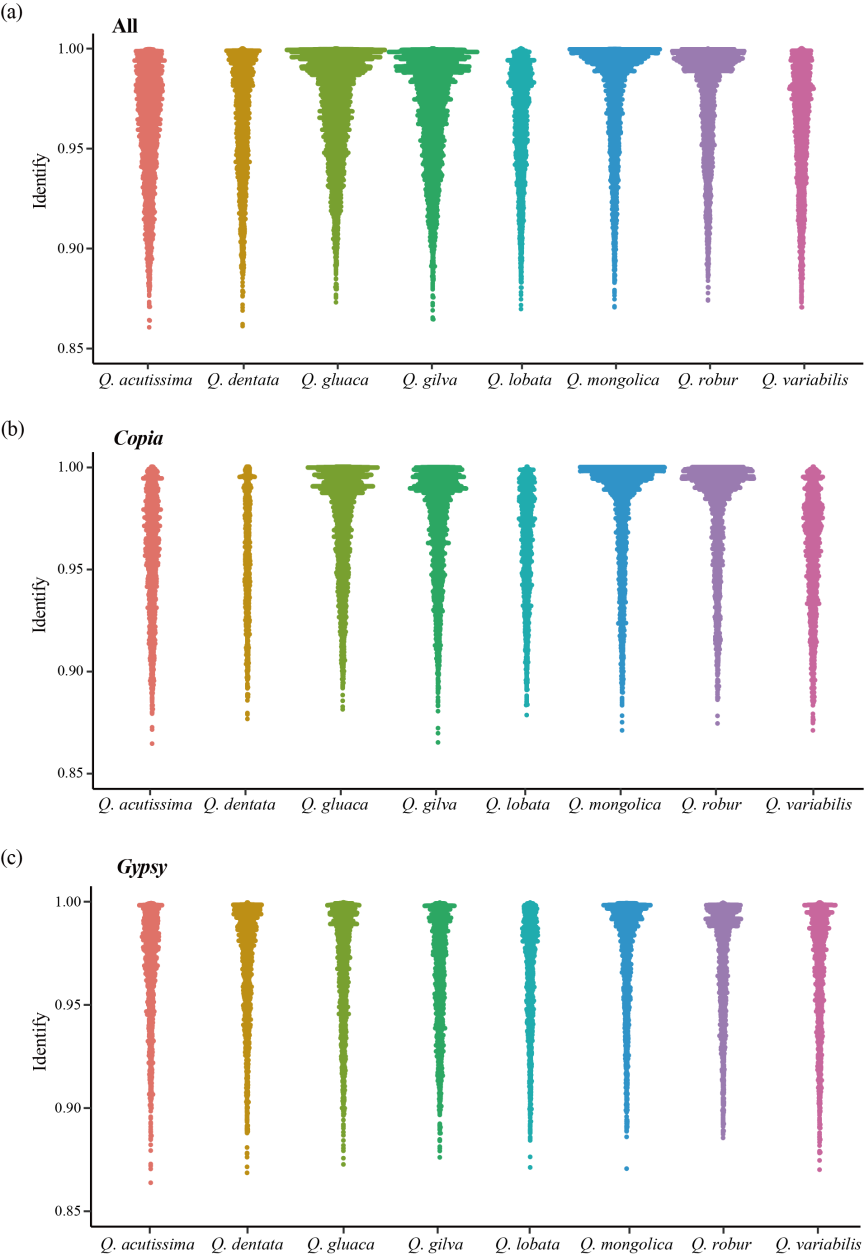


**Supplemental Fig. 6** Sequence identity distribution of LTR-RTs hits represented in a swarm plot for eight oak species. **(a)** Identity distribution of all elements; **(b)** Identity distribution of all *Copia*; **(c)** Identity distribution of all *Gypsy*.


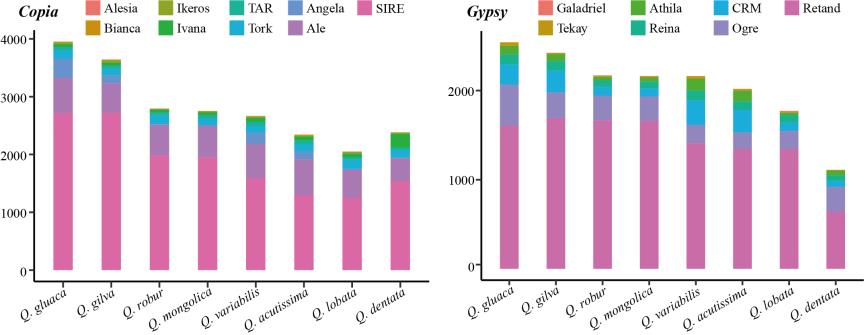


**Supplemental Fig. 7** Number of full-length LTR-RTs belonging to different lineages of *Copia* and *Gypsy* superfamilies.


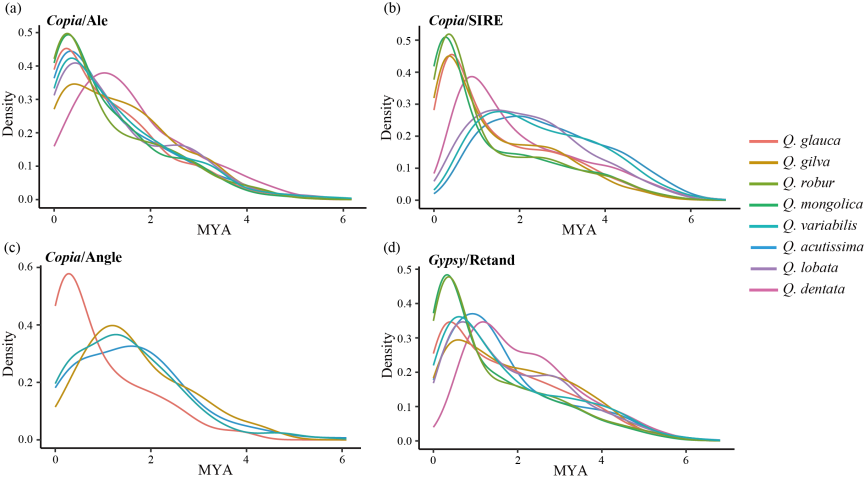


**Supplemental Fig. 8** Insertion time of the amplified LTR-RTs lineages in oak species (MYA, millions of years ago). **(a)** Insertion time of *Copia*/Ale; **(b)** Insertion time of *Copia*/SIRE; **(c)** Insertion time of *Copia*/Angela in *Q. acutissima,* *Q. gilva*, *Q. glauca*, and *Q. variabilis*; **(d)** Insertion time of *Gypsy*/Retand in all oak species.


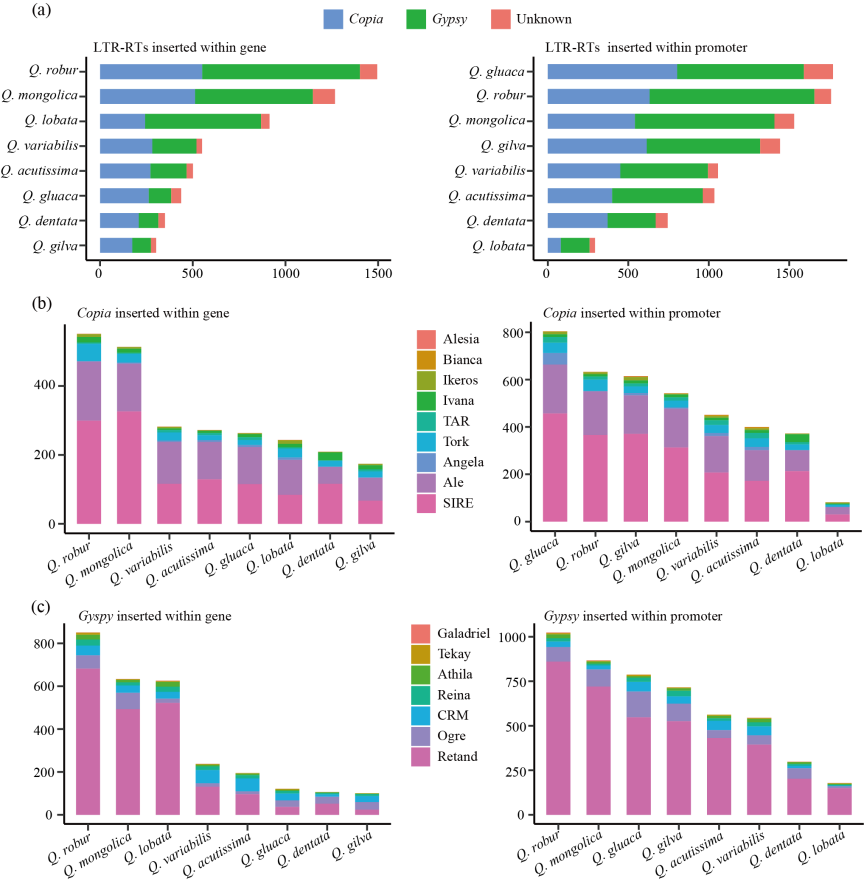


**Supplemental Fig. 9** Number of LTR-RTs inserted within gene and promoter. **(a)** Number of full-length LTR-RTs belonging to *Copia* and *Gypsy* superfamilies; **(b)** Number of different lineages of *Copia* superfamily; **(c)** Number of different lineages of *Gypsy* superfamily.


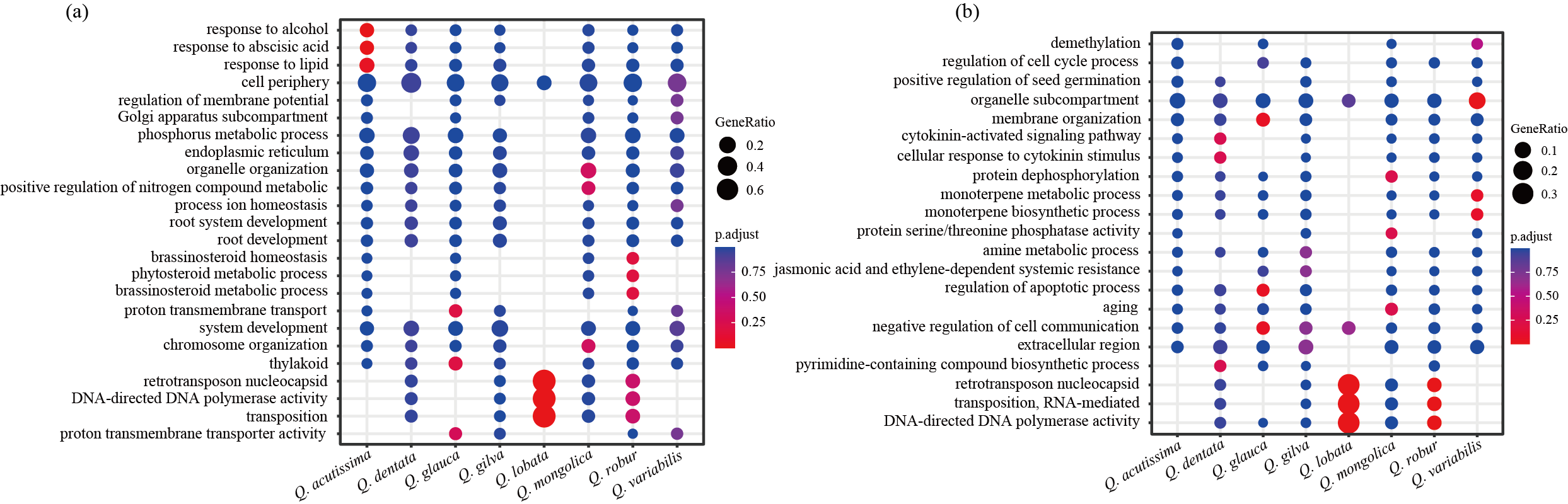


**Supplemental Fig. 10** GO enrichment analysis of LTR-RT-associated genes among eight oak species. **(a)** GO enrichment of LTR-RT-associated genes with LTR-RTs insertion in genes; **(b)** GO enrichment of LTR-RT-associated genes with LTR-RTs insertion in promoter regions.


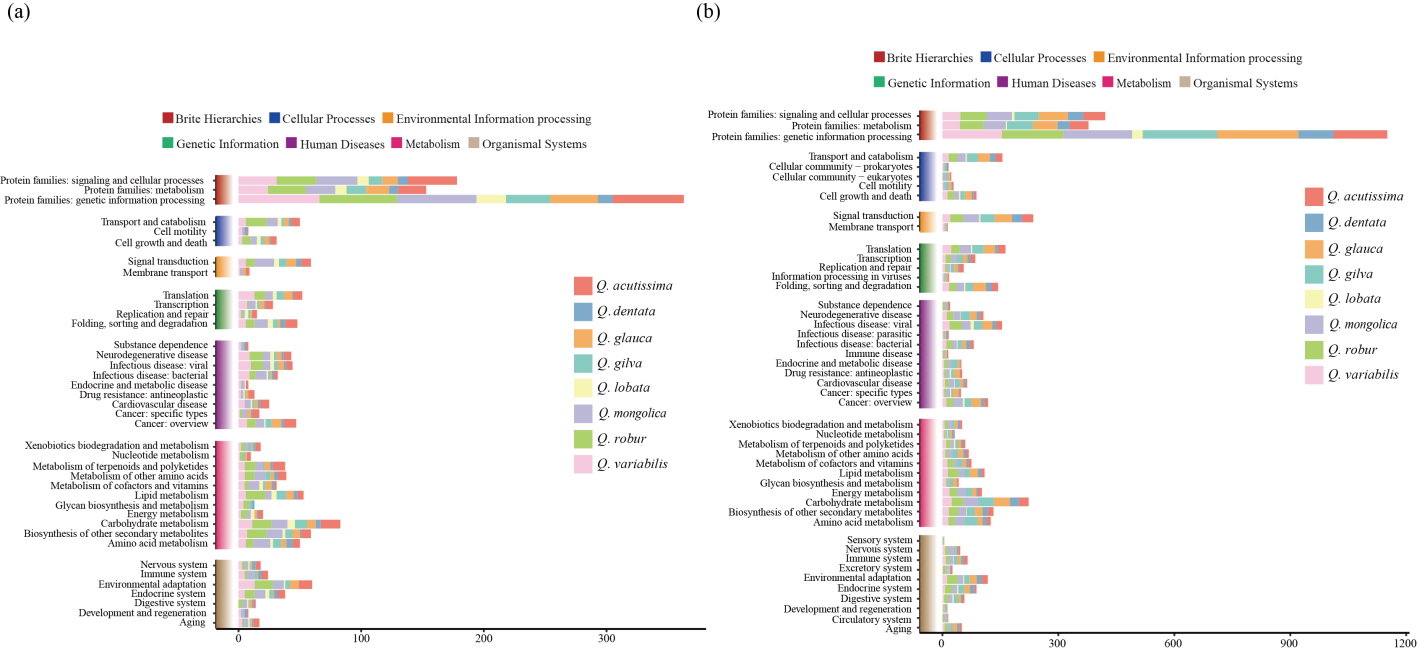


**Supplemental Fig. 11** KEGG enrichment analysis of LTR-RT-associated genes among eight oak species. **(a)** KEGG enrichment of LTR-RT-associated genes with LTR-RTs insertion in genes; **(b)** KEGG enrichment of LTR-RT-associated genes with LTR-RTs insertion in promoter regions.


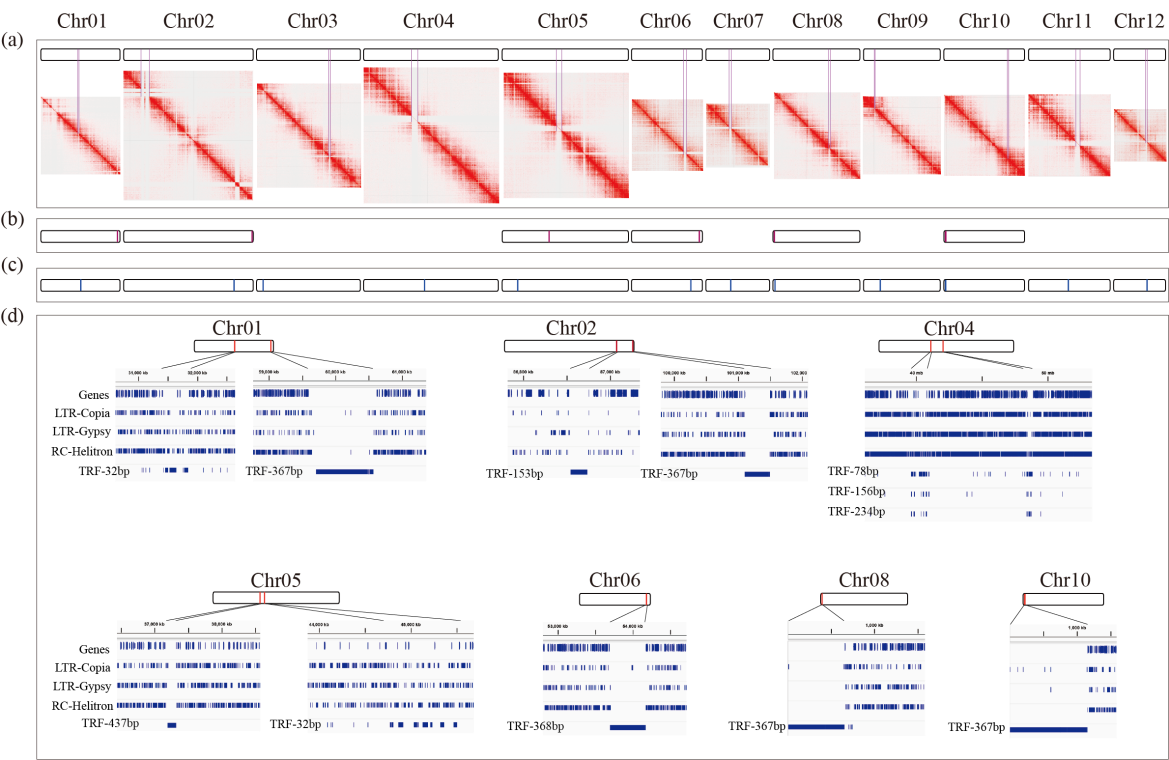


**Supplemental Fig. 12** Map of centromeres location information predicted by various methods. **(a)** Discontinuous signals in the chromatin interaction heat maps; **(b)** Centromere localization detection results of method Centromics; **(c)** Centromere localization detection results of CentroMiner tools of quarTeT; **(d)** High-frequency repeat units of Telomeres_and_Centromeres results.


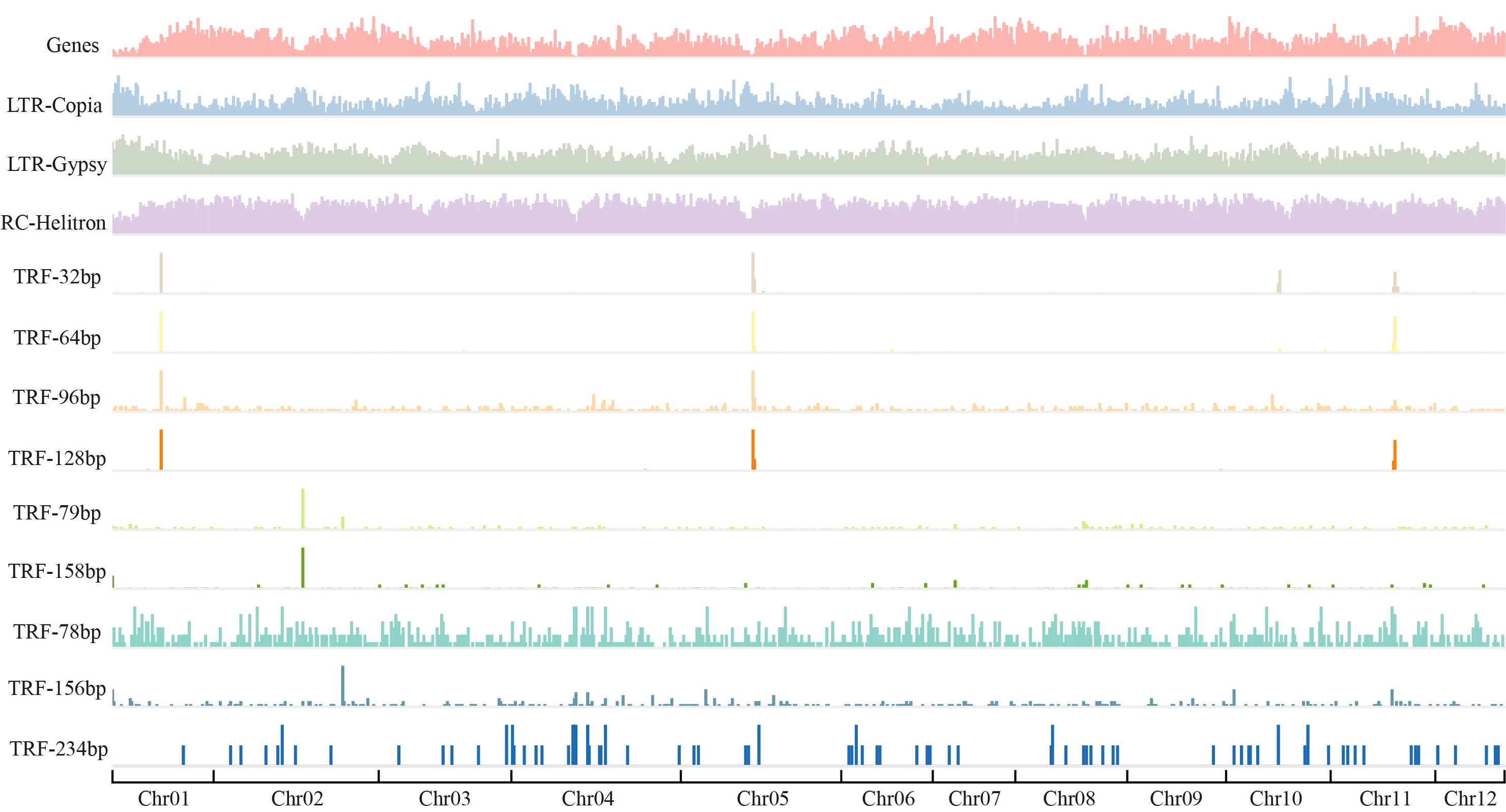


**Supplemental Fig. 13** Repeat annotation in *Q. glauca* reference genome.
